# GZMK^high^ CD8^+^ T effector memory cells are associated with CD15^high^ neutrophil abundance in non-metastatic colorectal tumors and predict poor clinical outcome

**DOI:** 10.1038/s41467-022-34467-3

**Published:** 2022-11-08

**Authors:** Silvia Tiberti, Carlotta Catozzi, Ottavio Croci, Mattia Ballerini, Danilo Cagnina, Chiara Soriani, Caterina Scirgolea, Zheng Gong, Jiatai He, Angeli D. Macandog, Amir Nabinejad, Carina B. Nava Lauson, Arianna Quinte’, Giovanni Bertalot, Wanda L. Petz, Simona P. Ravenda, Valerio Licursi, Paola Paci, Marco Rasponi, Luca Rotta, Nicola Fazio, Guangwen Ren, Uberto Fumagalli-Romario, Martin H. Schaefer, Stefano Campaner, Enrico Lugli, Luigi Nezi, Teresa Manzo

**Affiliations:** 1 Department of Experimental Oncology, European Institute of Oncology- IRCCS, Milano, Italy; 2 Center for Genomic Science of IIT, CGS@SEMM (Istituto Italiano di Tecnologia at European School of Molecular Medicine), Fondazione Istituto Italiano di Tecnologia (IIT), Milano, Italy; 3 Laboratory of Translational Immunology, IRCCS- Humanitas Research Hospital-, Milan, Italy; 4 The Jackson Laboratory, Bar Harbor, ME USA; 5 Department of Molecular & Biomedical Sciences, University of Maine, Orono, ME 04469 USA; 6 Department of Pathology, APSS Trento Hospital & Health Care, Trento, Italy; 7 Digestive Surgery Division, European Institute of Oncology-IRCCS, Milan, Italy; 8 Division of Gastrointestinal Medical Oncology and Neuroendocrine Tumors, European Institute of Oncology-IRCCS, Milan, Italy; 9 Institute of Molecular Biology and Pathology (IBPM), National Research Council (CNR) of Italy, Rome, Italy; 10 Department of Computer, Control and Management Engineering, Sapienza - University of Rome, Rome, Italy; 11 Department of Electronics, Information and Bioengineering, Politecnico di Milano, Milan, Italy

**Keywords:** Tumour immunology, Colorectal cancer

## Abstract

CD8^+^ T cells are a major prognostic determinant in solid tumors, including colorectal cancer (CRC). However, understanding how the interplay between different immune cells impacts on clinical outcome is still in its infancy. Here, we describe that the interaction of tumor infiltrating neutrophils expressing high levels of CD15 with CD8^+^ T effector memory cells (*T*_EM_) correlates with tumor progression. Mechanistically, stromal cell-derived factor-1 (CXCL12/SDF-1) promotes the retention of neutrophils within tumors, increasing the crosstalk with CD8^+^ T cells. As a consequence of the contact-mediated interaction with neutrophils, CD8^+^ T cells are skewed to produce high levels of GZMK, which in turn decreases E-cadherin on the intestinal epithelium and favors tumor progression. Overall, our results highlight the emergence of GZMK^high^ CD8^+^ T_EM_ in non-metastatic CRC tumors as a hallmark driven by the interaction with neutrophils, which could implement current patient stratification and be targeted by novel therapeutics.

## Abstract (web summary)

The tumor immune microenvironment is an important prognostic determinant in colorectal cancer (CRC). Here the authors show that tumor infiltrating neutrophils expressing high levels of CD15 interact with CD8^+^ T effector memory skewing them to produce GZMK, associated with tumor progression in CRC patients.

## Introduction

Colorectal cancer (CRC) is the third most commonly diagnosed tumor worldwide and a leading cause of cancer related death. Efficacy of treatment and survival are largely dictated by the tumor-node-metastasis (TNM) stage at diagnosis. Tumor contexture has emerged as a major prognostic determinant and a guide to implement therapy outcome^1,2^. However, 30–40% of surgically resected CRC relapse despite favorable TNM staging and significant lymphocyte infiltration^3^, become metastatic and patients ultimately die^4^, demonstrating the inadequacy of current stratification.

In this regard, cytotoxic CD8^+^ T cell infiltration has been associated with a better prognosis in CRC and other solid tumors^5,6^ and high levels of memory CD8^+^ T cells prevent early metastatic invasion and are associated with better survival^7^. However, the tumor immune infiltrate is highly heterogeneous and, although the influence of various myeloid cell populations on T cell activity has been reported^8–10^, the interplay between different cell compartments at the tumor site and their impact on the clinical outcome is just starting being exposed. In particular, tumor-associated neutrophils represent a significant fraction of the inflammatory cells in the tumor microenvironment (TME) of many types of cancers, including CRC^11–16^. The concept of neutrophil phenotypic and functional diversity in tumors has emerged from murine models^17^ and has been well documented in humans^18,19^, however, their contribution as pro- or anti-tumor factor remains inconclusive^20,21^.

Here, we show that CRC tumors highly infiltrated by neutrophils contain a population of CD8^+^ T_EM_ cells characterized by high levels of Granzyme K (GZMK^high^ CD8^+^ T_EM_ cells), which leads to worse prognosis.

## Results

### CRC patients can be stratified based on the abundance of CD15^high^ neutrophils in the tumor microenvironment

**Fig. 1:**
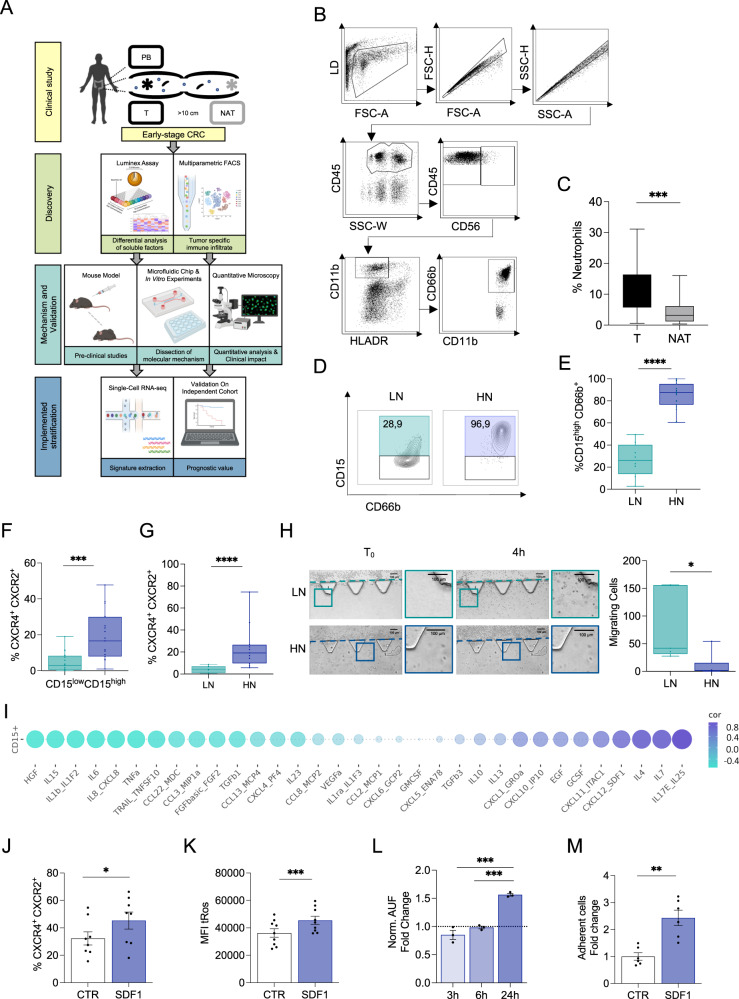
CD15^high^ neutrophils accumulate in a subset of non-metastatic resectable colorectal cancer (CRC) patients. **A** Study design created with BioRender.com. Analysis of Tumor tissue (T), Normal Adjacent Tissue (NAT), and Peripheral Blood (PB) of non-metastatic CRC patients (*n* = 46). **B** Flow cytometric gating strategy for the identification of CD15^high^ tumor infiltrating neutrophils. **C** Frequency of tumor infiltrating neutrophils defined as CD45^+^/CD11b^+^/HLADR^-^/CD56/CD66b^+^ within CD45^+^ cells in T vs NAT (*n* = 27, *p* = 0.0004). **D** Representatives contour plots of CD15 expression in low (LN) and high neutrophils (HN) patients. **E** Frequency of CD15^high^ cells in LN and HN patients (*n* = 8 LN and *n* = 19 HN, *p* < 0.0001). **F** Frequency of CXCR4^+^/CXCR2^+^ within CD15^low^ and CD15^high^ neutrophils (*n* = 19, *p* < 0.0001). **G** Frequency of CXCR4^+^/CXCR2^+^ within LN and HN patients (*n* = 7 LN and *n* = 11 HN, *p* = 0.0007). **H** Representative brightfield images of neutrophils motility assay on the microfluidic device. Interstitial Fluid (IF) of T from HN and LN patients was tested. Bar plot of number of migrating neutrophils on chip upon stimulation with IF from HN (*N* = 6) and LN (*N* = 5, *p* = 0.03) patients. Scale bar 100 μm. **I** Spearman correlation between soluble molecules in T IF and CD15^high^ neutrophils (*n* = 5). **J** Frequency of CXCR4^+^ CXCR2^+^ in HD SDF-1 treated^-^neutrophils (*n* = 9, *p* = 0.048). **K** Geometric mean of total ROS (tRos) MFI within HD fresh-isolated neutrophils treated with SDF-1 (*n* = 9, *p* = 0.0001). **L** Gelatinase activity assay on HD freshly isolated neutrophils treated with SDF-1 for 2 h, 6 h, and 24 h (*n* = 3). Bar plot showing fold change over untreated control (*p* = 0.2246 for 3 h vs 6 h, *p* = 0.0001 for 3 h vs 24 h, *p* = 0.0004 for 6 h vs 24 h). **M** Quantification of adherent neutrophils on endothelial cells after 2 h treatment with SDF-1 (*n* = 6, *p* = 0.0060). Bars represent mean ± SEM or box and whisker plots indicate Min to Max value, two tailed paired t test (**C**, **E**, **J**, **K**, **M**), two-tailed paired Wilcoxon test (**F**), two-tailed Mann–Whitney test (**G**–**H**), two-tailed one-way Anova (**L**). Source data are provided as a Source Data file.

Despite consistent T cell infiltration, over a third of CRC patients relapse, suggesting that the anti-tumor immune response is faulty. In this regard, characterizing the heterogeneity of immune cells within and across CRC patients would be essential to understand the mechanisms underlying immune evasion. We profiled peripheral blood (PB), tumor (T) and normal adjacent tissue (NAT, about 10 cm from tumor tissue) from treatment-naïve patients (*n* = 46 with non-metastatic resectable CRC undergoing surgery (Fig. 1A). Details of all patients involved in the study are summarized in Table 1. Among the infiltrating innate immune cell populations, dendritic cells (DCs) were unchanged, NK cells in lower proportion and macrophages (albeit representing less than 4%, Supplementary Fig. 1A) enriched in T compared to NAT. On the other hand, neutrophils—identified as CD45^+^CD56^−^CD11b^+^ HLA-DR^−^CD66b^+^ cells along with intermediate to low expression of CD33^22,23^ (Fig. 1B and Supplementary Fig. 1B)—were significantly enriched in T compared to NAT, both in terms of frequency (Fig. 1C) and density (CD66b^+^, Supplementary Fig. 1C). In particular, we distinguished two groups of patients based on the abundance of neutrophils expressing high levels of CD15^24^ (CD15^high^, Fig. 1D, E and Supplementary Fig 1D–G), namely LN (low CD15^high^ neutrophils, frequency <50%, 31,1% incidence in our cohort) and HN (high CD15^high^ neutrophils, frequency >50%, 68,9% incidence in our cohort). The multi-lobed nuclei and granular cytoplasm of FACS-sorted and GIEMSA-stained cells confirmed that both CD15^high^ and CD15^low^ were neutrophils (Supplementary Fig. 1H). Notably, CD15^high^ neutrophils were specifically enriched in HN tumors, while their frequency was similar both in the peripheral blood and NAT of LN and HN patients (Supplementary Fig. 1I), suggesting the existence of a tumor-specific regulation of CD15 expression on infiltrating neutrophils.

**Table 1:** CRC patients’ characteristics

Study Cohort		
	Count	Frequency (%)
Total of patients	46	100
Relapse	4	8.7
MSI	6	13.0
Age		
<50	2	4.4
50–70	23	50.0
>70	21	45.6
Sex		
F	21	45.6
M	25	54.4
Stage first diagnosis		
I	12	26.1
II	16	34.8
III	18	39.1
BMI		
Normal	19	41.3
Overweight	15	32.6
Obese	7	15.2
NA	5	10.9
Site of primary tumor		
Left	20	43.5
Right	24	52.2
Medium traverse colon	2	4.3
Tumor type		
Adenocarcinoma	43	93.5
Mucinous adenocarcinoma	3	6.5

Age, Gender, Stage at first diagnosis, Body Mass Index (BMI), Site of primary tumor, and Tumor type are shown for the study cohort.

To gain functional insights, we analyzed the co-expression of surface receptors CXCR2 and CXCR4, which play an antagonistic role essential for the neutrophil’s life cycle, regulating retention and release from the bone marrow^25,26^. It has been observed that neutrophils simultaneously expressing both CXCR2 and CXCR4 in the TME are associated with a N2-like tumor-promoting state^27,28^. We found that CXCR2^+^CXCR4^+^ cells were significantly higher within CD15^high^ neutrophils and HN tumors compared to the CD15^low^ neutrophils and LN tumors, respectively (Fig. 1F, G), while no differences were detected on neutrophils isolated from NAT (Supplementary Fig. 1J). CD15^high^ and CD15^low^ both expressed the maturation marker CD10, excluding an immature phenotype. However, CD15^high^ neutrophils expressed higher levels of CD10, which also distinguishes T-cell’s suppressive from stimulatory neutrophils (Supplementary Fig. 1K)^28,29^. Finally, neutrophils from HN tumors exhibited higher production of total reactive oxygen (tROS) species (Supplementary Fig. 1L), another pro-tumorigenic attribute.

Next, we wondered how CD15^high^ neutrophils accumulated in peripheral tissues. Neutrophil’s homeostasis results from the balance between macrophage-dependent clearance and recirculation, which is regulated by soluble factors^25,30^. Although the anti-correlation with macrophages (Supplementary Fig 1M, N) could link the higher abundance of CD15^high^ neutrophils in HN tumors to a lower clearance^31^, we did not detect any difference in the frequency of CXCR4^+^CXCR2^−^ cells (Supplementary Fig. 1O, P), excluding the enrichment of aged neutrophils^18^ in HN tumors. Thus, we assessed if the differential distribution of CD15^high^ neutrophils in HN and LN was driven by factors released in the TME. This was addressed by measuring the ability of freshly isolated neutrophils to migrate through a 3D collagen matrix on a custom-made microfluidic device. Surprisingly, neutrophils migrated more when exposed to interstitial fluids of LN compared to HN tumors (Fig. 1H), suggesting that HN tumors might impair their recirculation and cause accumulation. The increase in CD15 expression on neutrophils from healthy donors (HD) upon exposure to interstitial fluids of HN compared to LN tumors (Supplementary Fig. 2A) further supported an active role of the TME in driving neutrophil function and motivated the profiling of soluble factors in T and NAT from CRC patients. Out of 49 quantified molecules, 33 gave consistently measurable results (Supplementary Fig. 2B) and a subset was significantly correlated with the frequency of CD15^high^ neutrophils (Fig. 1I), independently from demographics or tumor grade. In particular, CD15^high^ neutrophils were positively correlated with IL-17E/IL-25, which plays important roles in gut inflammation^32–34^, it has been shown to upregulate the expression of CXCL1 and CXCL10^35^ and promotes a Th2-like responses by inducing IL-4, IL-5 and IL-13 expression^36^. In agreement, we found that also CXCL1, CXCL10, IL-4, and IL-13 were positively correlated with CD15^high^ neutrophils. Interestingly, IL-4/IL-13–stimulated neutrophils have been reported to have diminished ability to form NETs and migrate toward CXCL8^37^, suggesting that neutrophils from HN and LN might be functionally different. Enrichment of G-CSF and IL-10 in HN further supported that the TME drives neutrophil polarization toward a N2-like functional state^28,38–42^. Among all detected factors, CXCL12/SDF-1 (from now on referred to as SDF-1) was of particular interest because of its role in neutrophil trafficking and retention at inflammatory site through its binding to CXCR4^25,31^, which we reported to be highly expressed on CD15^high^ neutrophils together with CXCR2 (Fig. 1F, G). Imaging analysis confirmed that levels of SDF-1 were overall higher in HN compared to LN tumors and this was due to the combination of a larger number of αSMA^+^ cancer-associated fibroblasts and their higher expression of SDF-1 in HN (Supplementary Fig. 2C, D). However, while treatment with IL-8 and CXCL6 (two well-known chemo-attractants found to negatively correlate with CD15^high^ neutrophils in vivo) showed a pro-migratory effect, SDF-1 did not foster neutrophil’s mobility in vitro despite they expressed CXCR4 on the surface (Supplementary Fig. 2C, D). Instead, SDF-1 treatment induced higher frequency CXCR2^+^CXCR4^+^ double positive cells (Fig. 1J and Supplementary Fig. 2G, H), elevated level of total ROS (Fig. 1K) and increased gelatinolytic activity (Fig. 1L) and adhesion to micro-endothelial cells (Fig. 1M) compared to untreated controls, recapitulating part of in vivo observations. The slight increase in CD62L upon SDF-1 treatment supported again the idea that these neutrophils were not aged (Supplementary Fig. 2I)^31^. In summary, our data indicate that differences in the TME induce changes in the functional state of neutrophils and favor their retention in the tumor, leading to CD15^high^ neutrophils accumulation in a subgroup of patients.

### Two main CD8^+^ T cell populations identified by differential expression of CD39 and GZMK infiltrate CRC tumors

**Fig. 2:**
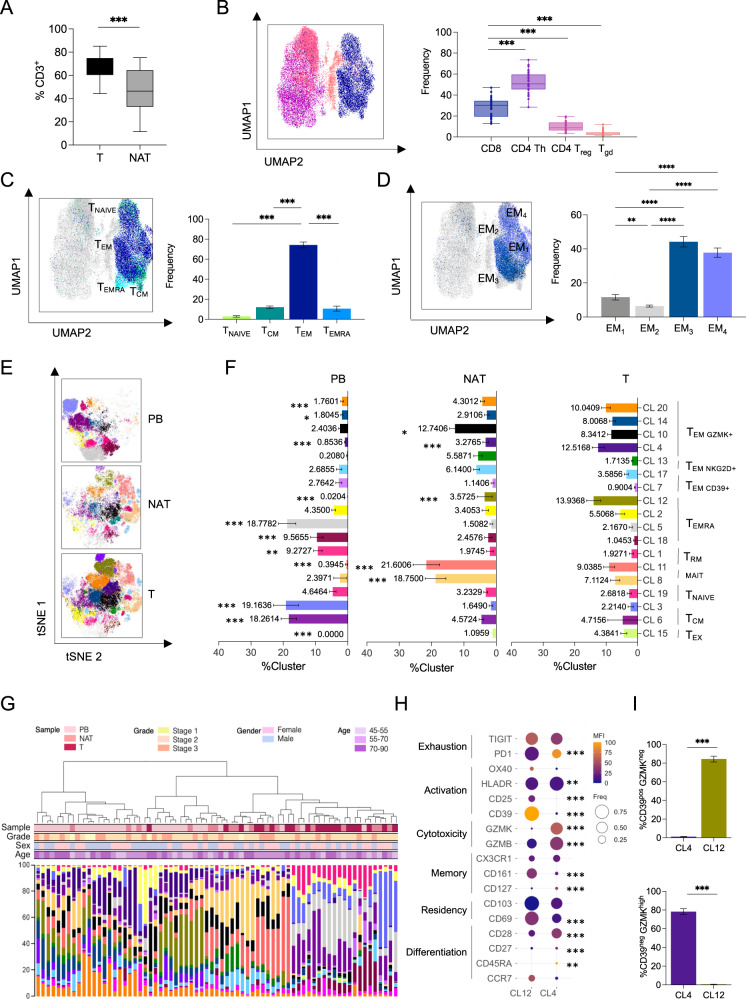
High-dimensional single cell analysis of CD8^+^ T cells identifies a tumor-specific CD39^neg^ GZMK^high^ population enriched in CRC. **A** CD3 Lymphocytes frequency within CD45^+^ in tumor (T) compared with normal adjacent tissue (NAT) (*n* = 29, *p* < 0.0001). **B**–**D** UMAP representation of concatenated CD3^+^ T cells from T samples with bar plot quantification. **B** Cytotoxic CD8^+^ T cells (blue), Th CD4^+^ (purple), Treg CD4^+^, CD25^+^, CD127^−^ (pink), and γδT cells (orange) distribution (*n* = 29, *p* < 0.0001). **C** Naive T cells (T_NAIVE_, light green), central memory T cells (T_CM_, dark green), effector memory T cells (T_EM_, dark blue), effector memory cells re-expressing CD45RA (T_EMRA_, light blue) distribution (*n* = 31) (*p* < 0.0001 for T_NAIVE_ vs T_CM_, T_NAIVE_ *vs* T_EM_, T_CM_ vs T_E_M, T_EM_ vs T_EMRA_; *p* = 0.0031 for T_NAIVE_ vs T_EMRA_; *p* = 0.9651 for T_CM_ vs T_EMRA_). **D** Effector memory T cells subtypes (EM_1–4_) (*n* = 31) (*p* < 0.0001 for EM_1_ vs EM_3_, EM_1_ vs EM_4_, EM_2_ *vs* EM_3_, EM_2_ vs EM_4_; *p* = 0.0071 for EM_1_ vs EM_2_; *p* = 0.6521 for EM_3_ vs EM_4_). **E** tSNE representation of Phenograph algorithm identified 18 clusters of concatenated CD8^+^ T cells from T (*n* = 34), NAT (*n* = 17), and PB (*n* = 22) from CRC patients. **F** Cluster representation in different tissues: PB, NAT, T. **G** Hierarchical metaclustering of CRC patient’s samples based on Phenograph identified clusters using Ward’s minimum variance method. For each sample, sample type, grade, sex, and age are indicated (*n* = 71). **H** Balloon plot of the average expression levels and expression frequencies of marker of differentiation, residency, memory, cytotoxicity, tumor reactivity, activation, and exhaustion in clusters identified in (**E**, **F**) and enriched in T (*n* = 34). **I** Frequency of CD39^pos^/GZMK^low^ and CD39^pos^/GZMK^high^ within Cluster 4 and Cluster 12 (*n* = 34) (*p* value <0.001). Bars represent mean ± SEM or box and whisker plots indicate Min to Max value, two-tailed paired Wilcoxon test (**A**), two-tailed one-way Anova (**B**–**F**), two-tailed paired t test (**I**). Source data are provided as a Source Data file.

We next hypothesized that the accumulation of CD15^high^ neutrophils might alter the immune landscape of the tumor, in particular CD8^+^ T cells. CD8^+^ T cell infiltration is unanimously accepted as a favorable prognostic marker in CRC^1,2,43–46^. However, querying the TCGA database of non-metastatic CRC patients with a cytolytic CD8^+^ T cell’s transcriptional signature did not result into an effective stratification (DFS, *p* = 0.12 Supplementary Fig. 3A), suggesting that other factors might influence CD8^+^ T cell-mediated responses and the clinical outcome. Therefore, we employed high dimensional flow cytometry to assess the diversity of the CD8^+^ T cell compartment, looking at markers of memory and effector differentiation (CD45RA, CCR7, CD27, CD28, CD127, CX3CR1, and CD161), activation (OX40, CD25, CD69, and HLA-DR), inhibitory receptors (PD-1 and TIGIT), tissue residency and tumor reactivity (CD103 and CD39) and effector molecules (GZMB and GZMK). The full list is reported in Table 2. CD3^+^ T cells represented over 60% of the immune infiltrate (Supplementary Fig. 3B) and were enriched in T compared to NAT across patients (Fig. 2A). In the T, the composition of the CD3^+^ T cells included CD4^+^ Th cells, CD8^+^ T cells, T regulatory (*T*_Reg_) cells and T gamma-delta (Tγδ) cells, with CD4^+^ Th and CD8^+^ T cells being predominant (Fig. 2B).

**Table 2:** Multiparametric flow cytometry panel

Marker	Fluorophore	Manufacturer	Cat. #
Zombie Aqua		BioLegend	423101
TCRgd	PerCP-Cy5.5	BioLegend	331224
NKG2A	FITC	Miltenyi	130-113-568
CD39	APC-H7	BioLegend	328226
TIGIT	APC	BioLegend	372706
CD25	BV786	BD	741035
CCR7	BV711	BD	566602
OX40	BV650	BD	563658
CD161	BV605	BioLegend	339916
CD27	BV570	BioLegend	302825
CD11b	BV510	BioLegend	301334
PD-1	BV480	BD	566112
CD103	BV421	BioLegend	350214
CD8	BUV805	BD	564912
CD28	BUV737	BD	564438
HLADR	BUV661	BD	565073
CD4	BUV615	BD	624297
CD45RA	BUV563	BD	565702
CD3	BUV496	BD	564809
CD69	BUV395	BD	564364
CD45	PE-Cy7	BioLegend	304016304016
CD56	PE-CY5.5	eBioscience	35-0567-42
CD127	PE-CY5	eBioscience	15-1278-42
CX3CR1	PECF594	BioLegend	341624
GZMB	APC-R700	BD	560213
GZMK	PE	Santa Cruz	sc-56125

Markers with the corresponding Fluorophore, Manufacturer, and Catalog ID (Cat. #) are shown.

Focusing on the CD8^+^ T cell compartment, naive (T_NAIVE_ identified by CD45RA^+^CCR7^+^) and central memory (T_CM_ identified by CD45RA^−^CCR7^+^) were rare both in the T and in NAT compared to PB (Supplementary Fig. 3C). In line with previous reports^7^, the majority (about 80%) of the infiltrating CD8^+^ T cells were effector memory (T_EM_), characterized by CCR7^−^CD45RA^−^ expression (Fig. 2C and Supplementary Fig. 3C). Based on differential CD27 and CD28 expression (Supplementary Fig. 3D), we further divided the T_EM_ into 4 phenotypically and functionally distinct subpopulations called EM_1_ (CD27^+^CD28^+^), EM_2_ (CD27^+^CD28^-^), EM_3_ (CD27^−^CD28^−^) and EM_4_ (CD27^−^CD28^+^)^47^. The T_EM3_ and T_EM4_ compartment, which represented effector-like and memory-like phenotypes respectively, were the most represented in T (Fig. 2D). We detected low frequencies of terminally differentiated effector memory cells re**-**expressing CD45RA (T_EMRA_)^48^ (Fig. 2C and Supplementary Fig. 3C) or exhibiting traits associated with exhaustion^49^, such as co-expression of PD-1, LAG3, and TIM3 (Supplementary Fig. 3E).

Analysis with t-distributed stochastic neighbor embedding (tSNE) analysis confirmed that CD8^+^ T cell profiles were substantially different in the PB, NAT, and T compartments also at single-cell level (Fig. 2E, F), independently from other factors like patient’s age, gender or tumor stage (Fig. 2G). Based on calculated mean fluorescence intensity (MFI) and frequency of different markers (Supplementary Fig. 3F), we identified 20 different clusters (CL) in the CD8^+^ T cell compartment (Supplementary Fig. 3G, H). CL 9 and 16 were excluded from further analyses because their frequencies were below 0.5^50–52^. A number of clusters were enriched in PB compared to NAT and T and were characterized by lack of expression of residency and activation markers (CD69, CD103, and CD39). These included: CL3 - annotated as naïve (T_NAIVE_) and characterized by high levels of CD45RA, CCR7, and CD127 expression and lack of activation markers; CL1, 5, and 18 were annotated as T_EMRA_ being characterized by low CCR7 level, general lack of expression of memory markers along with high levels of the cytolytic molecule GZMB; CL6 - annotated as central memory (T_CM_) because of the expression of several memory-associated markers (CCR7, CD127, CD27, and CD28) but absence of activation and cytotoxic markers (Fig. 2H and Supplementary Fig. 3G). Four other clusters, instead, were enriched in T and NAT compared to PB, indicating that they were tissue-infiltrating CD8^+^ T cell subsets. These were: CL14 and 20 mainly composed by CD45RA^neg^CCR7^neg^CD28^high^CD27^low^CX3CR1^pos^ T_EM_ cells, and CL8 and 11 annotated as tissue-resident memory T cells (T_RM_) since they displayed high expression of markers of memory (CD127 and CD28) and tissue residency (CD69 and CD103). These two tissue-infiltrating T_RM_ subsets expressed intermediate levels of CD161 and were mostly present in NAT (Fig. 2F and Supplementary Fig. 3G). In line with previous reports^53^, this population appeared to be different from the CD161^high^ mucosal-associated invariant T cells (MAIT) in CL19, which was equally represented in all three different compartments of CRC patients.

Since they were specifically and significantly enriched in T compared to NAT and PB, we next focused on CL12 and CL4, which were mainly composed by a T cell population reminiscent of CD8^+^ T_EM_ cells (CD45RA^low^CCR7^low^CD28^int^CD27^low^CD8^+^). They showed low or intermediate levels of CD127 and CX3CR1, and differential expression of activation, exhaustion, and cytotoxicity markers (Fig. 2H and Supplementary Fig. 3G). In particular, CL12 included CD8^+^ T cells with negligible expression of GZMK and high levels of CD39, a marker that has been recently associated with tumor antigen encounters^54–57^. On the contrary, CL4 was primarily composed by CD8^+^ T cells characterized by lack of CD39 and elevated levels of GZMK (Fig. 2I). Collectively, these data showed that CRC TME is mainly infiltrated by two distinct CD8^+^ T_EM_ populations that could be distinguished based on GZMK and CD39 expression (GZMK^high^CD39^neg^ and GZMK^low^CD39^pos^).

**Fig. 3:**
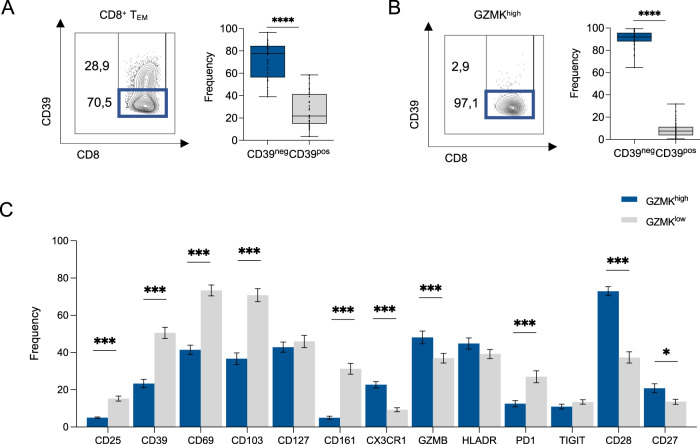
A CD39^neg^ and GZMK^high^ population characterizes CD8^+^ T cells infiltrating CRC. **A** Representative contour plot and quantification of CD39^neg^ and CD39^pos^ frequency within CD8^+^ T_EM_ (*n* = 34, *p* < 0.0001). **B** Representative contour plot and quantification of CD39^neg^ and CD39^pos^ frequency within GZMK^high^ CD8^+^ T_EM_. (*p* < 0.0001). **C** Expression level of indicated markers within GZMK^high^ and GZMK^low^ CD8^+^ T_EM_ (*p* = 0.0032 for CD25, *p* < 0.0001 for CD39, CD69, CD103, CD161, CX3CR1, PD-1, CD28, *p* = 0.3752 for CD127, *p* = 0.0012 for GZMβ, *p* = 0.1035 for HLA-DR, *p* = 0.4775 for TIGIT, *p* = 0.0362 for CD27. Bars represent mean ± SEM or box and whisker plots indicate Min to Max value, two-tailed paired Wilcoxon test (**A**, **B**), two-tailed unpaired multiple t test (**C**). Source data are provided as a Source Data file.

Using a manual gating strategy, we observed that CD8^+^ T_EM_ were predominantly CD39^neg^ (Fig. 3A), while the expression of CD103 and CD69 indicated that they were not derived from blood contamination (Supplementary Fig. 4A, B). Thus, we turned our attention on the characterization of the CD8^+^ T_EM_, annotated as CL4. Manual gating analysis showed that GZMK^high^ CD8^+^ T_EM_ cells were almost entirely CD39^neg^ (Fig. 3B), which distinguished them from the recently described “exhausted-like” GZMK^high^ CD8^+^ T cells^58–60^. Indeed, when compared to their GZMK^low^ counterpart, GZMK^high^ CD8^+^ T cells harbored the highest expression of activation markers (CX3CR1, HLA-DR, CD27, and CD28) and GZMB together with marginal expression of exhaustion markers (PD-1 and TIGIT) (Fig. 3C). When FACS-sorted based on CD39 expression, CD39^neg^ CD8^+^ T cells produced significantly higher levels of GZMK and retained the ability to produce IFNγ, TNFα and GZMβ compared to their CD39^pos^ counterparts (Supplementary Fig. 3D). In conclusion, the TME of CRC patients is highly infiltrated by GZMK^high^ CD8^+^ T_EM_ cells, which lack CD39 expression.

### CD8^+^ T_EM_ expressing GZMK have a distinct transcriptional program and correlate with worse outcome

To better characterize infiltrating GZMK^high^CD8^+^ T_EM_ cells and define their prognostic potential, paired T and NAT tissues from 8 HN patients were profiled by single cell RNA sequencing (scRNAseq). We collected data from a total of 52316 cells. Mean reads per cell were 94183 (min:37500; max:235694) with a median number of genes detected per cell of 3779 (min:1433; max:5392). Sequencing saturation was >49% for all samples, indicating comprehensive sampling of the available transcripts. After processing and normalization, dimensionality reduction was performed with uniform manifold approximation and projection (UMAP). Shared nearest neighbor (SNN) clustering distinguished eleven different clusters that were annotated to nine broad cell lineages, including epithelial (*EPCAM*^*+*^), endothelial (*CD34*^*+*^), mesenchymal (*MMP2*^*+*^), B cells (*MZB1*^*+*^), and T/NK (*CD3D*^*+*^) (Supplementary Fig. 5A–C). Notwithstanding the expected variability among patients, all the annotated clusters were represented across the patients (Supplementary Fig. 5D). Expression analysis across all the annotated cell subtypes highlighted the T/NK cluster as the main source of *GZMK* expression in the TME of HN (Supplementary Fig. 5E).

**Fig. 4:**
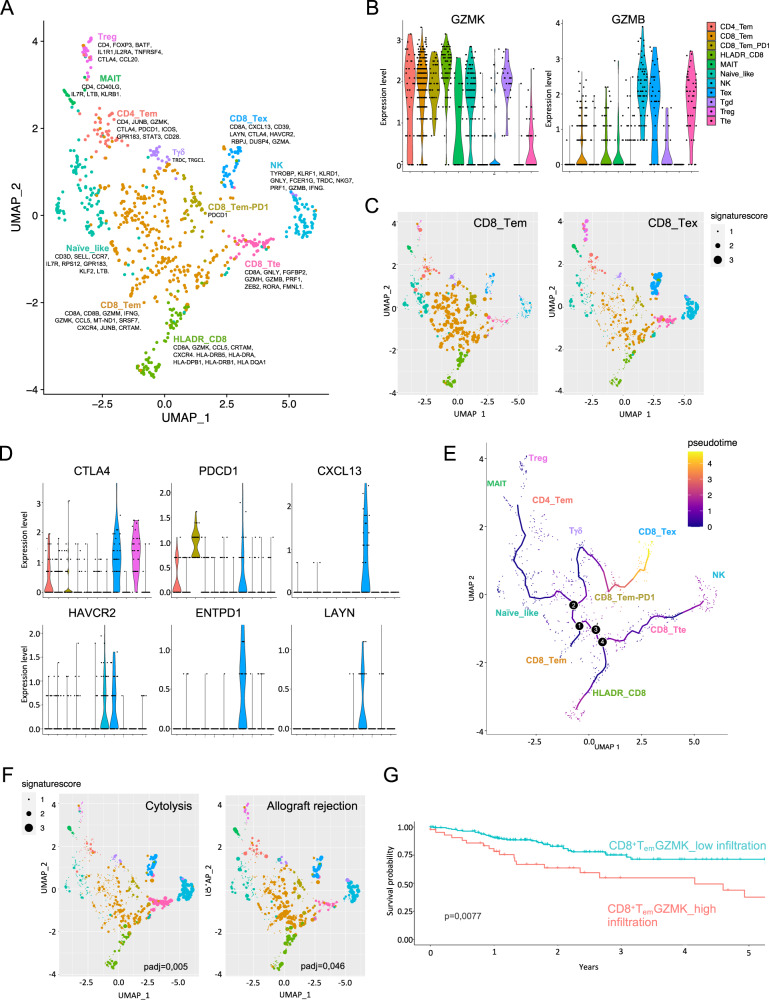
Single-cell RNAseq analysis on CRC patients defines an effective prognostic signature based on CD8_T_em_ infiltration. **A**–**F** scRNAseq analysis on 8 CRC patients. **A** UMAP projection of CD8^+^ T cells. Colors represent different T cell subtypes. Key genes used for manual annotation are indicated. **B** Violin plots showing normalized expression of *GZMB* and *GZMK* in the different T CD8^+^ cell subtypes. **C** UMAP showing the expression of CD8_Tem and CD8_Tex signatures for each cell. Cells are color coded based on SNN-clustering, dot’s size reflects the expression of the signature for each cell. **D** Violin plots showing the expression of genes associated with T-cell exhaustion within the indicated cell subtype. Color code as in (**B**). **E** Monocle pseudotime analysis of the CD8^+^ T cell subtypes. The pseudotime line connects the indicated T cell subtypes identified in (**A**). **F** UMAP as in (**C**) showing the expression of Cytolysis and Allograft-rejection signatures. **G** Kaplan–Meyer analysis of the association of CD8_T_em_ cell subtype’ abundance with disease-free survival (DFS) on the TCGA-COAD cohort (*n* = 284; see “Methods” and Supplementary Table 1 for details). High infiltration in red, low infiltration in turquoise.

Zooming in on the 8523 cells composing the T/NK cluster, we generated a new UMAP that further distinguished seven T cell subtypes (Supplementary Fig. 5F), among which cluster 1 was enriched in CD4^+^ while clusters 3 and 4 were enriched in CD8^+^ T cells (Supplementary Fig. 5G, H). Interestingly, *GZMK* expressing cells were mostly restricted to the CD8^+^ clusters 3 and 4, confirming results from our phenotypic analysis. Within the CD8^+^-enriched T cells clusters 3 and 4, we could identify eleven sub-clusters (Fig. 4A). These clusters were annotated with manually curated gene-sets (Supplementary Table 2) based on literature^61^.

While confirming the presence of previously described putative T cell sub-populations, this analysis highlighted a remarkable transcriptional heterogeneity. The eleven clusters included minor fractions of CD4^+^ T cells falling in the memory (CD4_T_EM_), the *FOXP3* expressing regulatory (Treg), *CD40LG* positive mucosal-associated invariant T (MAIT) and exhausted (Tex) T cell clusters. Co-expression of *TRDC* and *TRGC1* enabled discrimination of gamma delta T cells (Tγδ), while higher expression of *AKR1C3*, *CXC3R1*, *FCER1G*, *NKG7,* and *PRF1* were distinctive transcriptional traits of the natural killer (NK) cluster. Naive-like T cells were assigned based on the expression of *IL7R*, *CCR7,* and *SELL* signature genes (Supplementary Fig. 6A, C). Most of the retrieved T cells fell within the CD8^+^ T effector memory subgroup (CD8_T_em_, Supplementary Fig. 6B) characterized by high expression of *GZMK* (Fig. 4B) in agreement with those identified by FACS analysis. On the other hand, differential analysis of the CD8_T_em_ and CD8_T_ex_ signature and marginal expression of genes associated to exhaustion (namely *CTLA-4*, *PDCD-1*, *CXCL13*, *HAVCR2*, *ENTPD1,* and *LAYN*) revealed that CD8_T_em_ were considerably distinct from T_ex_ cells (Fig. 4C, D). In addition, pseudotime analysis showed that CD8_T_em_ were closer to Naive-like T cells than to terminally differentiated effectors (T_te_, Fig. 4E and Supplementary Fig. 6D). The borders between CD8_T_em_ and the clusters labeled as CD8_T_em_-PD1 and HLADR_CD8 were diffuse, instead, even though hierarchical clustering of differentially expressed genes (DEGs) confirmed the robustness of the model. While suggesting that transcriptional gradients might contribute to T cell heterogeneity, this observation also marked intermediate states on the transition of CD8_T_em_ toward exhaustion, for example, through the expression of genes like *PDCD1* and *CXCL13*. Functionally, pathway enrichment analysis indicated that, despite having lower cytolytic potential (padj = 0.005) compared to T_te_ and NK cells, both CD8_T_em_ and HLADR_CD8 (both expressing high levels of *GZMK* levels) were not impaired in proliferation (Supplementary Fig. 6E). In particular, CD8_T_em_ had lower expression of lymphocyte activation and migration signatures and higher cell-cell adhesion signatures (Supplementary Fig. 6E). Moreover, subgrouping of the CD8_T_em_ based on *GZMK* expression (*GZMK*^neg^ and *GZMK*^pos^, Supplementary Fig. 6F) showed similar levels of the effector molecule *TNFA* in the two subsets (Supplementary Fig. 6H), confirming that *GZMK* expression was not associated with a dysfunctional state (see also Supplementary Fig. 3D). When we searched for transcription factors (TF) within CD8_T_em_’s DEGs, we found three annotated TF or cofactor that could drive their state, namely *RORA*, *JUNB* and *HOPX* (Supplementary Fig. 6I). In particular, *RORA* was downregulated specifically in the CD8_T_em_ and the HLADR_CD8 subsets, in agreement with its association with effector function and cytotoxicity rather than memory^62^ while the antithetical expression of *JUNB* (a subunit of *AP-1*, upregulated) and *HOPX* (an *AP-1* cofactor, downregulated) was in agreement with their role as regulators of *AP-1* function^63^ and might suggest loss of the transcriptional brake that suppresses acute T cell responses preventing tissue damage^64^.

Finally, querying of publicly available CRC datasets on The Cancer Genome Atlas (TCGA) using CD8_T_em_ transcriptional signature (see Methods for details) showed a better prognosis in patients with lower infiltration of CD8_T_em_ expressing *GZMK* (Fig. 4G) and this was specific of the CD8_T_em_, since none of the other lymphocyte signatures tested resulted into an equivalent stratification (Supplementary Figs 7B–D and Supplementary 8). Importantly, our CD8_T_em_ was effective in stratifying also patients with other types of carcinomas, like the lung adenocarcinoma (LUAD on TCGA, Supplementary Fig. 7E).

Overall, functional and transcriptional characterization of the tumor immune infiltrates led to an effective stratification of patients and predicted the risk of encountering secondary events in CRC patients and, potentially, in other cancer types.

### Neutrophils directly interact with infiltrating CD8^+^ T cells increasing GZMK expression

**Fig. 5:**
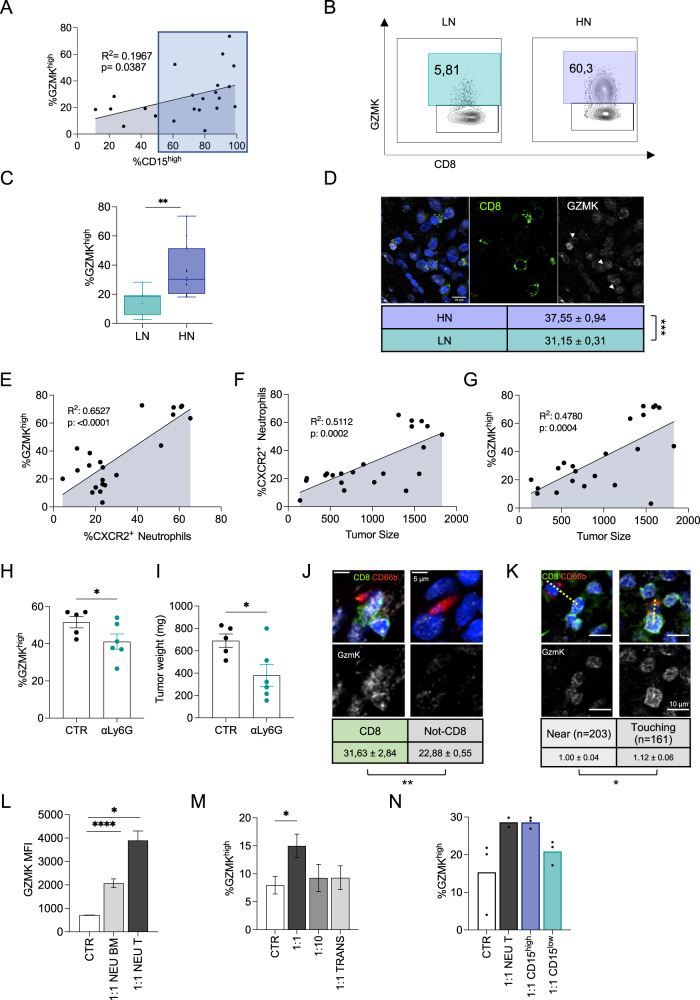
Contact-mediated interaction of neutrophils with CD8^+^ T cells induces GZMK expression. **A** Pearson correlation between CD15^high^ neutrophils and GZMK^high^ CD8^+^ T_EM_ (*n* = 22; *p* = 0.0387) **B** Representative contour plots of GZMK expression in CD8^+^ T_EM_ in LN and HN patients. **C** Frequency of GZMK^high^ CD8^+^ T_EM_ cells in LN (*n* = 7) and HN (*n* = 14, *p* = 0.009) patients. **D** GZMK Mean Fluorescence Intensity (MFI) in HN (*n* = 9) and LN (*n* = 8) tumors. Scale bar 10 μm. Pearson correlation between: CXCR2^+^ neutrophils and GZMK^high^ CD8^+^ T cells (**E**, *n* = 22, *p* < 0.0001); CXCR2^+^ neutrophils and tumor size (**F**, *n* = 22, *p* = 0.0002), GZMK^high^ CD8^+^ T cells and tumor size (**G**, *n* = 21, *p* = 0.0004) within tumor (T) of MC38 tumor-bearing mice. GZMK^high^ CD8^+^ T frequency within T (**H**, *p* = 0.050) and tumor weight (**I**, *p* = 0.030) in mice upon αLy6G antibody-mediated neutrophil’s depletion (*n* = 6) or treatment with isotype control (*n* = 5). **J** Representative confocal images and quantification of GZMK MFI in CD8^+^ and CD8^−^ (not-CD8) cells within a 10 μm distance from neutrophils (CD66b^+^). Scale bar 5 μm. **K** Representative confocal images and normalized quantification (see “Methods”) of GZMK MFI in CD8^+^ within 10 μm (Touching, *n* = 161) or between 10 and 20 μm (Near, *n* = 203) from neutrophils (CD66b^+^). Yellow dotted lines represent 20 μm (left) and 10 μm (right). Scale bar 10 μm. **L**. GZMK^+^ MFI within activated CD8^+^ T cells (CTR) co-cultured at 1:1 ratio with neutrophils isolated from Bone Marrow (BM, *n* = 9) or MC38 tumors (T, *n* = 3) (*p* = 0.015 for CTR *vs* 1:1 NEU T, *p* < 0.0001 for CTR *vs* 1:1 NEU BM. **M**. Frequency of GZMK^high^ CD8^+^ T cells (CTR) after co-culture with HD neutrophils (*n* = 16), either in plate or in Transwell (TRANS). The CD8^+^ T cells-neutrophils ratios are indicated (*p* = 0.011 for CTR vs 1:1, *p* = 0.098 for CTR vs 1:10, *p* = 0.976 for CTR vs 1:1 TRANS). **N**. Co-culture of CD8^+^ isolated from patients’ PB with neutrophils isolated from tumor (NEU T) (*n* = 2), CD15^high^ and CD15^low^neutrophils. Bars represent mean ± SEM or box and whisker plots indicate Min to Max value, two-tailed paired t test (**C**, **L**, **M**, **N**), two-tailed Mann–Whitney test (**H**), two-tailed unpaired t test (**I**). Source data are provided as a Source Data file.

It is well accepted that neutrophils influence CD8^+^ T cell function, but there is not general consensus on their effect on T cell-mediated responses, yet^18,21,65–69^. While combining overall neutrophils with the cytolytic CD8^+^ T cell transcriptional signatures did not independently predict the clinical outcome (Supplementary Fig. 9A, *p* = 0.44), Pearson correlation analysis revealed that intratumoral CD15^high^ neutrophils were positively correlated with GZMK^high^ CD8^+^ T_EM_ (Fig. 5A), which we have shown to effectively stratify CRC and LUAD patients. As expected, GZMK^high^ CD8^+^ T_EM_ were also positively correlated with CXCR2^+^ and CXCR4^+^ neutrophils (Supplementary Fig. 9B, C), further supporting a specific cross-talk with intratumoral CD15^high^ neutrophils, which was not seen when considering other CD8^+^ T cell differentiation states or activation markers (Supplementary Fig. 9D, E). Since CD4^+^ T cells were the predominant population in the CD3^+^ T cell compartment, we sought to address their potential contribution to GZMK production. Our data showed that CD4^+^ T cells produced low amounts of GZMK (Supplementary Fig. 9F), confirming scRNAseq results, and they were neither correlated with CD15^high^ neutrophils abundance (Supplementary Fig. 9G) nor differentially represented in the TME of HN and LN (Supplementary Fig. 9H). In line with decreased frequencies of macrophages in HN tumors, we also found that GZMK^high^ CD8^+^ T_EM_ cells were anti-correlated with macrophages (Supplementary Fig. 9I). Together, these data prompted us to investigate the specific cross-talk between CD15^high^ neutrophils and GZMK^high^ CD8^+^ T_EM_ cells.

In agreement with previous reports^70–72^, GZMK^+^CD8 T cells were detected - albeit at low frequencies - in the peripheral blood of HD and CRC patients as well as in NAT of CRC patients at the same extent. However, intratumor CD8^+^ T_EM_ cells expressing higher levels of GZMK specifically and significantly accumulated only in HN T compared to their corresponding NAT (Supplementary Fig. 9J), which supports the idea that the GZMK^+^ immune signature we found is tumor-specific.

Importantly, FACS (Fig. 5B, C and Supplementary Fig. 9J) and quantitative confocal microscopy (Fig. 5D) independently confirmed a higher proportion of GZMK^high^ CD8^+^ T_EM_ cells in HN tumors.

To validate the interaction of neutrophils with GZMK^high^ CD8^+^ T_EM_ on an independent system and to address its contribution to the anti-tumor response, we analyzed the immune infiltrate during tumor progression on the syngeneic MC38 mouse model of colon cancer^50^. Similar to what we observed in patients, intratumoral murine GZMK^high^ CD8^+^ T cells were positively correlated with CD11b^+^Ly6g^+^CXCR2^+^ intratumoral neutrophils (Fig. 5E). Moreover, both CXCR2^+^ neutrophils and intratumoral GZMK^high^ CD8^+^ T_EM_ positively correlated with tumor size (Fig. 5F, G), supporting the existence of a GZMK^high^ CD8^+^ T_EM_ -neutrophils crosstalk that might favor tumor progression. Indeed, in vivo neutrophils depletion (as assessed in bone marrow, blood, spleen, and tumors, Supplementary Fig. 9K) significantly reduced intratumoral frequencies of GZMK^high^ CD8 T cells (Fig. 5H) paralleled by a reduction in the tumor size (Fig. 5I), demonstrating that the GZMK^high^ CD8^+^ T_EM_-neutrophils crosstalk is conserved across species and contributes to tumor progression. However, experimental evidence of a direct influence of neutrophils on the GZMK^high^ CD8 T cell phenotype was missing, still. First, we showed by quantitative confocal microscopy on human CRC tumors that the contact (distance *r* ≤ 10 µm) with intratumoral neutrophils (CD66b^+^) was associated with levels of GZMK in CD8^+^ T cells higher than any other cell types (not-CD8^+^, Fig. 5J), confirming that the interaction is specific and possibly contact-mediated. Thus, we employed a similar approach to demonstrate that CD8^+^ T cells expressed higher levels of GZMK when in contact (Touching, *r* ≤ 10 µm) with neutrophils compared to close proximity (Near, 10 µm ≤ *r* ≤ 20 µm) (Fig. 5K).

We independently confirmed these data by co-culturing activated CD8^+^ T cells with neutrophils from mice (Fig. 5L) or humans (Fig. 5M), which led to an increased frequency of GZMK^high^ CD8^+^ T cells in a cell ratio- and physical contact-dependent manner. Importantly, the expression of GZMK by CD8^+^ T cells was induced to a larger extent upon co-culturing them with neutrophils isolated from tumors compared to either PB or bone marrow (BM) in mice (Fig. 5L), reinforcing the idea that the TME plays a pivotal role in this cross-talk. We obtained similar results when using human cells, with a greater increase in GZMK expression by CD8^+^ T cells induced by tumor-derived neutrophils (Fig. 5N) compared to neutrophils from PB (Fig. 5M). Importantly, coculturing of CD8^+^ T cells with CD15^high^ neutrophils FACS-sorted from human HN tumors produced higher GZMK levels compared to CD15^low^ (Fig. 5N), indicating that CD15^high^ neutrophils are the key players in inducing GZMK production on CD8 T cells.

Overall, these studies confirm the existence of a direct, contact-mediated cross-talk between neutrophils and CD8^+^ T cells resulting into increased GZMK production, and support a model where GZMK^high^ CD8^+^ T cells might foster tumor progression.

### The GZMK produced by infiltrating CD8^+^ T_EM_ promotes relapse in CRC

**Fig. 6:**
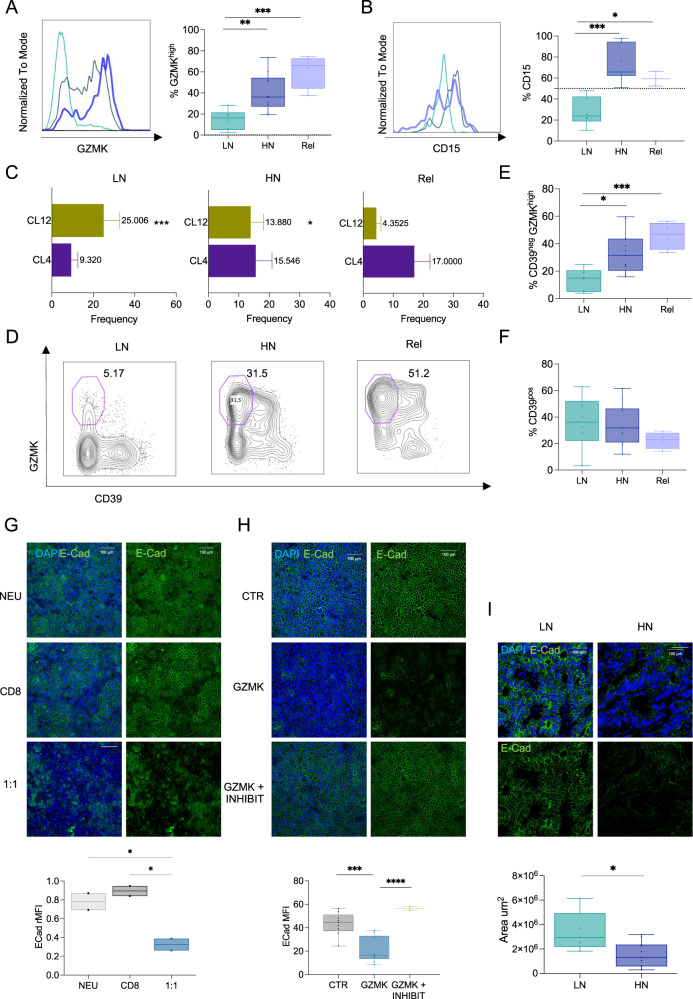
GZMK produced by infiltrating CD8^+^T_EM_ is associated with early relapse in CRC. **A**, **B** Histogram and quantification of GZMK (**A**; *p* = 0.005 for LN vs HN, *p* = 0.0004 for LN vs Rel, *p* = 0.076 for HN vs Rel)) or CD15 (**B**; *p* = 0.0003 for HN vs LN, *p* = 0.027 for LN vs Rel, *p* = 0.329 for HN vs Rel) in LN (*n* = 6), HN (*n* = 10) and early-relapsed (Rel, *n* = 4) patients. **C** Bar plot of Cluster 12 (CL12, green - *p* = 0.0022 for HN vs LN, *p* = 0.0151 for HN vs Rel, *p* < 0.0001 for LN vs Rel) and Cluster 4 (CL4, purple - *p* = 0.0854 for HN vs LN, *p* = 0.7093 for HN vs Rel, *p* = 0,0832 for LN vs Rel) in LN (*n* = 10), HN (*n* = 5) and Rel (*n* = 4). **D** Representative contour plot of GZMK and CD39 expression within CD8^+^ T_EM_ in HN, LN, and Rel patients. Frequency of CD39^neg^ GZMK^high^ (**E**; *p* = 0.011 for HN vs LN, *p* = 0.0005 for LN vs Rel, *p* = 0.157 for HN vs Rel) or CD39^pos^ (**F**; *p* = 0.858 for HN vs LN, *p* = 0.160 for HN vs Rel, *p* = 0.238 for LN vs Rel) in LN (*n* = 6), HN (*n* = 11) and Rel (*n* = 4). **G**, **H** Representative images and quantification of E-Cadherin expression on: **G** CACO-HT29 co-culture on transwells in presence of neutrophils (NEU), CD8 or neutrophils/CD8 (1:1) isolated from HD (*n* = 2, *p* = 0.3371 for NEU vs CD8, *p* = 0.0191 NEU *vs* 1:1, *p* = 0.0104 for CD8 *vs* 1:1); **H** CACO-HT29 treated with PBS (CTR) or recombinant active human GZMK, with (GZMK + INHIBIT) or without (GZMK) GZMK’s inhibitor (*n* = 10, *p* = 0.0001 for GZMK *vs* CTR, *p* < 0.0001 for GZMK vs GZMK + INHIBIT, *p* = 0.18 for CTR vs GZMK + INHIBIT). DAPI blue, E-Cadherin in green. Scale bar 100 μm. **H** Representative images and quantification of Scale bar 100 μm. **I** Representative images and quantification of E-Cadherin expression by multicolor confocal imaging FFPE sections from LN (*n* = 5) and HN (*n* = 12, *p* value = 0.0140) CRC tumors. DAPI blue, E-Cadherin green. Scale bar 100 μm. Bars represent mean ± SEM or box and whisker plots indicate Min to Max value, two-tailed unpaired t test (**A**, **B**, **E**, **F**, **I**), two-tailed two-way Anova (**C**), two-tailed one-way Anova (**G**, corrected with Fisher’s LSD test, **H**). Source data are provided as a Source Data file.

**Fig. 7:**
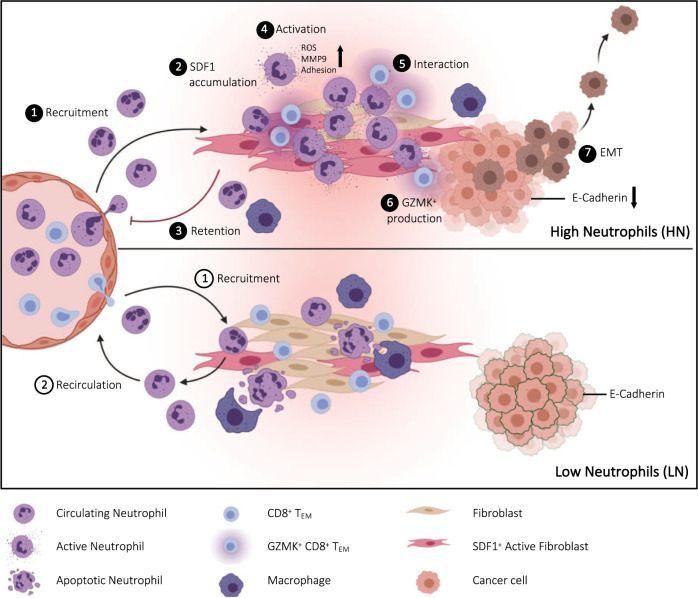
Graphical model. Abundance of CD15^high^ neutrophils in the tumor identifies High (HN) and Low (LN) neutrophil subgroups of non-metastatic resectable CRC patients (1). Elevated SDF-1 levels in HN tumors reshape the functional state of infiltrating neutrophils (2), promoting their retention (3) and activation (4) at the tumor site. The contact-mediated interaction with neutrophils (5) pushes effector memory CD8^+^ T cells (T_EM_) to produce high levels of Granzyme K (GZMK) (6), which in turn remodels the tumor microenvironment (TME) fostering EMT (7). A GZMK^high^ T_EM_ transcriptional signature effectively stratify non-metastatic resectable CRC patients and predict poor clinical outcome. Graphical model created with BioRender.com.

We observed that T from patients that encountered early relapse (Rel, *n* = 4) had frequencies of CD15^high^ neutrophils and GZMK^high^ CD8^+^ T_EM_ cells similar to HN and significantly higher than LN (Fig. 6A, B). They were enriched in CL4 (mainly composed of CD8^+^ T_EM_ GZMK^high^CD39^neg^) while CL12 (mostly CD8^+^ T_EFF_ GZMK^low^CD39^pos^) was poorly represented (Fig. 6C–E). Of note, frequencies of CD39^pos^ were comparable in LN, HN and Rel (Fig. 6F), indicating that the higher frequency of GZMK^high^CD39^neg^ observed in relapsed tumors was not due to a lower frequency of tumor-reactive CD39^pos^ T cells. Thus, we sought to elucidate the mechanistic details linking GZMK production by CD8^+^ T_EM_ to tumor relapse. Emerging evidence suggests that GZMK might promote inflammation and untargeted tissue damage^73^. This has been related to the activity of GZMK as an extracellular protease and its ability to promote epithelial-to-mesenchymal transition (EMT) by remodeling components of the extracellular matrix^74^. We tested this hypothesis in vitro by exposing a functional human intestinal epithelial model to the neutrophils-CD8^+^ T cells co-culture, which we have shown to induce GZMK production by CD8^+^ T cells. Compared to the co-culturing with each of the two cell types alone, the co-culture with both cell types induced a significant decrease in E-Cadherin expression (Fig. 6G), a well-known feature associated to EMT and tumor malignancy^75,76^. This result was not due to the previously reported pro-apoptotic effect of intracellular GZMK^74^, as demonstrated both by similar cancer cell cellularity and viability in the epithelial monolayers (Supplementary Fig. 10A, B). Similar results were obtained when the intestinal epithelial model was treated directly with recombinant human GZMK (Fig. 6H and Supplementary Fig. 10C, D), providing evidence that release of GZMK would be sufficient to induce tissue remodeling. As predicted, tumors from HN patients showed reduced expression of E-Cadherin compared to LN (Fig. 6I), suggesting that an enhanced release of GZMK by CD8^+^ T_EM_ upon interaction with intratumoral neutrophils promotes tumor progression by impacting the integrity of surrounding tissue, which ultimately contribute to relapse (Fig. 7).

## Discussion

We conducted unbiased analysis of the immune cells within and across CRC patients by combining multiparametric flow cytometry with functional assays and single cell transcriptomics. Our data reveal that CRC patients can be stratified based on high (HN) or low (LN) CD15^high^ neutrophil infiltration and highlight the emergence of a GZMK^high^ CD8^+^ T_EM_ cell population as a hallmark of HN tumors. The predominance of the GZMK^high^ CD8^+^ T_EM_ cells in CRC patients that encountered an early relapse and the correlation with poor clinical outcome on an independent cohort underline the clinical relevance of the cell population and its therapeutic potential.

Despite several attempts to profile the immune cell content of CRC^7,58^, the prognostic value of immune cell infiltration is not conclusive, especially regarding neutrophils^77^. We provide evidence that neutrophils together with NK cells are the most abundant innate cell population infiltrating the tumor bed in resectable CRC. Pro-tumorigenic roles have been ascribed to neutrophils across different tumor types^78,79^, including promotion of angiogenesis and immunosuppression^23,80^, but it is not clear whether distinct neutrophil populations gather the tumor or if phenotypic changes occur inside the TME. The presented analysis of the canonical neutrophil marker CD15^24^ reveals phenotypic changes specifically in the population associated with tumors. Intratumor neutrophils from LN patients have significantly lower expression of CD15 compared to healthy- and patient-derived peripheral neutrophils. Conversely, neutrophils infiltrating the TME of HN patients maintain high levels of CD15 expression, are enriched in CXCR2^+^CXCR4^+^ cells and tend to produce more ROS, in line with a so called N2-like tumor-promoting functional state^28^. Interestingly, treatment of freshly isolated neutrophils with interstitial fluid from HN tumors led to higher CD15 expression but, differently from interstitial fluid from LN, did not foster migration, suggesting that specific soluble factors in the TME might polarize neutrophils toward a CD15^high^ state and retain them within the tumor. However, the low yield of neutrophils FACS-sorted from human tumors based on CD15 expression limited our ability to compare CD15^high^ and CD15^low^ migration in vitro, which will require further investigation. Neutrophils are recruited early to the site of inflammation, where their non-specific antimicrobial functions can endanger the integrity of the host tissue in case of inappropriate retention^81,82^. To avoid it, in addition to clearance by macrophages-directed efferocytosis^30,83^, neutrophils redistribute into surrounding tissues or vasculature through reverse migration^84–87^, which has been suggested to follow desensitization to local chemotactic gradients^85,88,89^. In this regard, SDF-1/CXCR4 signaling plays an important role in the retention of neutrophils at inflammatory sites^90^. Since clearance by resident tissue macrophages has been associated with the reduction of CD15 surface expression on neutrophils^91^, we speculate that the enrichment of CD15^high^ neutrophils in HN tumors might be due, at least in part, to inefficient clearance, as previously demonstrated in other tumor contexts^31^. Indeed, we described an inverse correlation between macrophages and CD15^high^ neutrophils abundance, suggesting lower neutrophil’s clearance rate in HN CRC tumors. A question that warrants further study is which type of macrophages, M1 or M2, are responsible for neutrophils retention in NH tumors. Moreover, a detailed characterization of the nature and dynamic of CD15^low^ in relationship with CD15^high^ neutrophils in healthy individuals other than patients remains open and would highly contribute to the debate on the heterogeneity of human neutrophils.

On the other hand, our analysis of differentially abundant soluble factors pointed at SDF-1, which correlates in vivo with frequency of CD15^high^ neutrophils and, in vitro, increased adhesion and release of TME’s remodeling factors rather than driving chemotaxis. Overall, these data suggest that elevated concentrations of SDF-1 may promote the reprogramming of neutrophils infiltrating HN tumors. Future studies would need to confirm how this may lead to increased retention of neutrophils in the tumor, altering their homeostatic turnover.

Neutrophils influence CD8^+^ T cell-mediated responses both in animal models and humans. However, results are still sparse and often conflicting^18,21,65–69^. We provide evidence that direct interaction between neutrophils and CD8^+^ T cells leads to increased GZMK expression within CD8^+^ T cells, both in vivo and in vitro. When compared to their GZMK^neg^ counterpart, GZMK^high^ CD8^+^ T_EM_ cells harbor significantly lower expression of co-inhibitory receptors, higher levels of activation markers and effector molecules like GZMβ. Moreover, their abundance in the TME is independent from tumor stage and, importantly, from aging (*P* > 0.05, ANOVA)^60^. Thus, the identified GZMK^high^ CD8^+^ T_EM_ cell population cannot be considered as CD8^+^ T cells with an exhausted phenotype, as the ones recently described^58–60,92^ and that other mechanisms besides T cell exhaustion may contribute to the failure of tumor control.

Tumor-specific CD8 T cells could be distinguished from virus-specific CD8 T cells based on the expression of specific surface protein, in particular CD39 and PD-1^55,56^, which have previously been associated with antitumor responses^57,93^. It is worth it to emphasize that the GZMK^high^ CD8^+^ T_EM_ cell population enriched in HN lacked CD39 expression, suggesting that they are not specifically directed towards tumor-associated antigens^57,93^ but rather towards irrelevant antigen^55,56^. Since they have been previously associated with inflammatory disease^94,95^, the high proportion of GZMK^high^ CD39^neg^ CD8^+^ T_EM_ cells found in CRC patients raises the possibility that they might mediate a cross-reactive response and favors cancer progression. This model is supported by our transcriptomic analysis, which revealed that GZMK^high^ CD8^+^ T_EM_ cells harbored a peculiar alloreactive-like state that might be controlled by *RORA*, *JUNB,* and *HOPX*. While downregulation of *RORA* fits with the memory phenotype^62^, *HOPX*-dependent downregulation of *FOS* and *JUN* maintain a state of nonproliferation upon rechallenge^63^. In CD8^+^ T cells, this state of unresponsiveness can develop either in absence or following activation in the presence of costimulation, like on cells responding to a tumor^96,97^ and it has been linked to mechanisms of suppression of acute T cell responses^98^ where it downregulates and prevents immunopathologic damage to the tissue in which it occurs^64^. Therefore, the downregulation of *HOPX* in concert with the upregulation of *JUNB* found in GZMK^high^ CD8^+^ T_EM_ would be consistent with their persistent acute activation, which may lead to tissue damage through the release of GZMK. Future analysis addressing TCR clonality of GZMK^high^ CD39^neg^ CD8^+^ T_EM_ will allow testing this hypothesis unequivocally.

We show that, at least in HN CRC patients, CD8^+^ T_EM_ are the main source of GZMK. Further studies are needed to determine whether CD8^+^ T cells recruited to the tumor and skewed by neutrophils toward a GZMK^high^ phenotype also impacts neutrophil homeostasis and function, as already proved for other immune cell populations^99^. Regardless of the order of events, GZMK production by CD8^+^ T cells might play a pathogenic role by contributing to inflammation and tissue damage. This relates to its non-cytotoxic activity as extracellular protease and its ability to promote cellular migration and epithelial-to-mesenchymal transition (EMT) by remodeling components of the extracellular matrix^73,100^. In line with this, HN tumors showed reduced expression of E-Cadherin compared to LN, which is a feature of EMT and tumor malignancy. However, along with E-Cadherin attenuation, upregulation of other well-known EMT-associated markers (i.e., N-cadherin, TWIST, SNAI1-2) remains to be investigated to dissect the downstream molecular pathways modulated by GZMK.

Along with tangible therapeutic implications for future interventions, high expression of GZMK by infiltrating CD8^+^ T_EM_ in response to CD15^high^ neutrophils links them to tumor progression rather than anti-tumor function, suggesting that integrating functional information resulting from the crosstalk of CD8^+^ T cells with different components of the immune contexture and the stroma might implement the prognostic value of current biomarkers, which are mostly based on limited phenotypic or transcriptional CD8^+^ T cell characteristics.

## Methods

### Ethics approval for the research

The use of human samples was approved by the European Institute of Oncology (IEO) Institutional Review Board (protocol n. R1083/19-IEO 1149). T, NAT, and PB were collected from patients diagnosed with CRC. All patients were non-metastatic and treatment-naïve at the time of surgical resection (Table 1 includes all the relevant patient’s information). The collection of samples from healthy donors has been approved by the IEO’s Ethical Committee (registered as IEO 1781). All donors provided written informed consent in accordance with the Declaration of Helsinki.

Mice were housed and bred in a specific-pathogen-free animal facility and treated in accordance with the European Union Guideline on Animal Experiments. Animal handling and experimental protocols were reviewed and approved by the Institutional Animal Care and Use Committee of The Jackson Laboratory under the protocol number AUS#17027.

For mouse experiments, we used age-matched (10 weeks) C57BL/6J male mice in each experiment. The number of animals (biological replicates) was indicated in the respective figure legends.

### Cell lines

Caco-2 were obtained from American Type Culture Collection (ATCC). HT-29 MTX cells were a gentle gift from Dr. Monteleone (University of Rome Tor Vergata, Italy). Caco-2 were cultured in flasks in Eagle’s minimal essential medium (EMEM, Merck) supplemented with FBS, HEPES, sodium pyruvate, non-essential amino acids, L-Glutamine. HT-29 MTX were cultured in DMEM supplemented with FBS and HEPES. For co-culture experiments, Caco-2 and HT-29 MMTX were cultured in Caco-2 medium. Human Microvascular Endothelial Cells (HMEC-1) were obtained from Center for Disease Control and Prevention (CDC) of Atlanta. HMEC-1 were cultured in collagen-coated flasks in MCBD 131 medium supplemented with FBS, HEPES, Hydrocortisone and human epidermal growth factor (EGF). MC38 cell line was a gift from Dr. Chih-Hao Chang lab at The Jackson Laboratory. MC38 cells were maintained in DMEM supplemented with 10% fetal bovine serum (FBS). Cells were cultured in a humidified environment at 37 °C in 5% CO_2_.

### Tissue dissociation and cell isolation

Primary tumors (T) and Normal Adjacent Tissue (NAT, collected at about 10 cm from the tumor site) were collected and processed immediately after surgery, which was always scheduled in the morning to reduce circadian bias. Briefly, tissues were minced in cold PBS and incubated on ice to allow sedimentation of cellular debris. The supernatant (Interstitial fluid) was collected and spinned at 1200 g at 4 **°**C and then used fresh or stored at −80 °C. Interstitial fluid was prepared from T and NAT. Mechanical and enzymatic disaggregation (DNase I 2 mg/ml, Sigma; Collagenase 0.02 mg/ml, Roche) was used to obtain single-cell suspension. Samples were then filtered through 70-μm cell strainers and washed in PBS without Ca^2+^ and Mg^2+^.

Peripheral blood mononuclear cells (PBMCs) were isolated via density-gradient separation with Ficoll. Total CD8^+^ T cells were enriched by negative magnetic separation using the Miltenyi CD8^+^ T Cell Isolation Kit Human. Human polymorphonuclear leukocytes (PMNs) enriched pellets were diluted with PBS and 3% dextran solution in HBSS was added in a 1:1 ratio. After centrifugation, the supernatant was processed for red blood cell lysis according to manufacturer’s instructions (Red Blood Cell Lysis Solution, Miltenyi). Cells were either used fresh or cryopreserved according to standard procedures. Neutrophils, instead, were always used fresh in order to minimize technical artifacts and to maximize reproducibility. Mouse neutrophils and CD8^+^ T cells were purified according to manufacturer’s instructions using anti-Ly6G and CD8^+^ T Cell Isolation Kit Mouse, respectively (Miltenyi Biotech) or by FACS sorting. Purity of isolated human and mouse immune populations were checked by FACS (>95%).

### In vitro CD8^+^ T cells and neutrophils co-culture

CD8^+^ T cells activated with plate-bound anti-human CD3 2μg/mL (BD) and anti-human CD28 2μg/mL (BD) and freshly isolated neutrophils were co-cultured at different ratios in complete RPMI-1640 media.

### In vitro GZMK and SDF1 treatments

HT-29 and Caco-2 cells were cultured in transwells in a ratio 1:9 for 16 days, thus trans-epithelial electrical resistance (TEER) was measured to assess the epithelial formation. Cells were treated with rHu-GZMK (Enzo Life Sciences) 10 μM for 24 h, alone or in combination with GZMK inhibitor (PRO-328 100 nM, Prospec). For treatment of neutrophils, freshly cells isolated from PB of HD were treated with rHu-GZMK 10uM or SDF1 100 ng/ml (R&D biosystems) and analyzed at different time points as indicated in the relative figure legend.

### Neutrophils migration assay

Neutrophil migration assays were performed on neutrophil isolated from peripheral blood of healthy donors using a custom-made microfluidic device in PDMS composed by three parallel channels (height 200 µm, length 1.1 cm, width central channel 1300 µm, lateral channels 700 µm) separated by two lines of trapezoidal pillars. The central channel was used to confine a collagen matrix gel, the right channel for cell injection and the left channel for treatments. PBS 10×, NaOH 1N, 8.04 mg/ml rat tail Collagen type I (Corning) and RPMI culture medium (without phenol red) were mixed on ice to obtain a collagen matrix solution. The collagen solution was injected in the central channel of the microfluidic chip and incubated at 37 °C to allow the complete reticulation of the collagen matrix. The collagen matrix was hydrated by injecting culture medium into the lateral channels of the device. Purified human neutrophils (3 × 10^6^/ml) from healthy donor were labeled with calcein-AM (cat. C3100MP, Invitrogen), Hoechst 33342 (cat. BK4082S, Euroclone), and DRAQ7 (cat. BOS-DR71000, Vinci-Biochem) and injected in the right channel of the microfluidic devices. The microfluidic chips were incubated at 37 °C for 20 min on an angled surface (30 degrees) to allow neutrophils to lie at the interface between the lateral channel and the collagen gel. Chemoattractant solutions or culture medium were added to the left channel and time lapse experiments were performed using Nikon Eclipse Ti (3 fields for chip, 1 image every 2.30 min for 4 h at 10× magnification) to evaluate invasion of neutrophils in the collagen gel. Interstitial fluid from patients’ tissues, purified CXCL6 (400 ng/ml), SDF-1 (100 ng/ml) and IL-8 (100 ng/ml) (R&D biosystems) were tested. Migrating cells were counted and analyzed using ImageJ version 2.0.

### Neutrophils gelatinase assay

The gelatinase activity was measured from control or SDF1 (100 ng/mL) treated neutrophils in triplicate, using DQ gelatin (molecular Probes, 100 µg/mL final concentration) following manufacturer’s recommendations. The fluorescent output was measured every 15 min at room temperature on a GloMax Discover (Promega Instrument) microplate reader (Ex = ~495 nm, Em = ~515 nm) and corrected for background. *Clostridium histolyticum* collagenase (0.15 U/mL) was used to generate an internal calibration curve.

### Neutrophils adhesion assay

Purified human neutrophils were labeled with calcein-AM calcein (1:1000) e Hoechst 33342 (1:10,000) for 20 min at 37 °C^101^. After washing, neutrophils were incubated in RPMI media with or without SDF1 (100 ng/mL) for 1 h at 37 °C in a 96-well on a monolayer of human micro-endothelial cells (HMEC1). After two washes with PBS to remove non-adherent cells, images of three fields were recorded for each well using an EVOS microscope (EVOS FL Cell Imaging System) at 4× magnification. Adherent neutrophils were measured by counting calcein positive neutrophils using ImageJ version 2.0.0.

### Multiparametric flow cytometry and sorting

High-dimensional flow cytometry was performed on T, NAT, and PB^51^. Conjugated antibodies used for flow cytometry are shown in Table 2. Briefly, single-cell suspensions were stained fresh or after thawing in pre-warmed RPMI with 10% FBS. Cells were washed in staining buffer (PBS with 2%FBS and 2 mM EDTA) and incubated for 30 min at 4 °C with surface antibody cocktail in staining buffer, washed twice in staining buffer, fixed and permeabilized using Fixation/Permeabilization Solution Kit (BD, Cat # 554714), and stained intracellularly for 1 h at 4 °C with an antibody cocktail in the permeabilization solution. Samples were acquired using a FACSymphony A5 or a FACSCelesta BVYG equipped with FACSDiva software version 8.0.1 (all from BD Biosciences). Compensation Beads (ThermoFisher, Cat# 01-2222-41) were used to prepare single-stained controls for electronic compensation. Dead cells were excluded using Fixable Viability Stain (BD). To evaluate ex vivo cytokines production, CD8^+^ T cell subsets were sorted to high purity using FACSAria III (BD Biosciences), then stimulated 3 h with PMA (20 ng/mL), ionomycin (1 μg/mL) and GolgiPlug protein-transport inhibitor (brefeldin A, 1:1000).

All the FCS files generated were analyzed and visualized with software FlowJo v10 (TreeStar). RPhenograph^102^ package was used to analyze the multiparametric data from CRC patients derived T, NAT and PB. CD8 analysis was performed on 3000 events per sample after manual gating isolation of singlet, LD negative, CD45^+^CD3^+^CD8^+^ T cells. All samples were concatenated by the “cytof_exprsMerge function”. The number of nearest neighbors identified in the first iteration of the algorithm, *K* value, was set to 100. UMAP and tSNE representation were generated and visualized using FlowJo version 10 (FlowJo). Under-represented clusters (<0.5%) were discarded in subsequent analysis. The balloon plots, performed using the ggplots2 R package, generate a further metaclustering classification, interpolating MFI and frequency of each marker per cluster. MFI were multiplied to derive the integrated MFI (iMFI, rescaled to values from 0 to 100). Euclidean distance and Ward-linkage methods via cytofkit package were applied to generate hierarchical metaclustering of Phenograph generated clusters. For the neutrophils analysis the following gating strategy was applied: Live, CD45^+^, CD56^−^, HLA-DR^−^, CD11b^+^, CD33^dim^, CD66b^+^ and then selected the CD15^+^ and CD15^−^ populations. In order to optimize the multicolor panel used to identify different neutrophil’s subpopulations, first we tested an unstained control, the FMO for CD15 in CD66b^+^ neutrophils and the CD15 staining in a biological negative control, which were all negative compared to the CD15 staining in CD66b^+^ neutrophils (Supplementary Fig. 1D). Therefore, we adopted NK cells (defined as CD11b^+^, CD66b^−^, CD56^+^) and M-MDSC (defined as CD11b^+^, HLA-DR^−^, CD66b^−^, CD33^+^) as negative controls for CD15 expression in the following analysis. Neutrophils isolation from tumor samples was performed using a FACSAria cell sorter using the gating strategy described before. In addition to the high dimensional multiparametric flow cytometry panel shown in Table 2, we included all the other used antibodies in Supplementary Table 1.

### Bead-based multiplexed ELISA

Multiplexed ELISA on interstitial fluid from matched T and NAT fresh tissues were performed on a Luminex 200 platform (Luminex Inc.,) using custom kits of pre-mixed antibody-coated beads (R&D System Inc., MN), which included the following analytes: CCL11_eotaxin, CCL13_MCP4, CCL17_TARC, CCL2_MCP1, CCL22_MDC, CCL26_EOTAXIN3, CCL5_RANTES, CCL8_MCP2, CD25_IL2Ra, CX3CL1_FRACTALKINE, CXCL1_GROa, CXCL10_IP10, CXCL11_ITAC1, CXCL13_BLC_BCA1, CXCL4_PF4, CXCL5_ENA78, CXCL6_GCP2, EGF, IFNγ, GMCSF, HGF, IL10, IL1ra_IL1F3, IL7, IL8_CXCL8, TRAIL, VEGFA, IL1b_IL1F2, IL5, IL6, IL-17F, IL22, IL23, TNFα, LXSAHM-01, CCL3_MIP1a, CCL4_MIP1b, FGFbasic_FGF2, GCSF, IL1a_IL1F1, IL2, IL4, IL-12 p70, IL13, IL15, IL-17/IL17A, CXCL12_SDF1, TGFB1, TGFB3. The assay was performed based on manufacturer recommendations. Briefly, 50 µl of samples together with kit standards were added to each well in duplicate and incubated with the diluted Microparticle Cocktail at 4 °C, ON, on a shaker at 850 rpm. Unbound soluble molecules were removed by washing the plate. The Biotin-Antibody Cocktail specific to the analytes of interest was added to each well for 1 h at RT. After washing again, the Streptavidin-Phycoerythrin conjugated was added for 30 min at RT. After the final washing steps, the microparticles are resuspended in kit buffer and read on a Luminex 200 platform. The outputs (pg/mL) were visualized and statistically analyzed in R upon centering and scaling using the scale function in R (SD from mean pg/mL). Data were visualized together with sample annotations using the ComplexHeatmap package. Group comparisons were visualized as boxplots using the ggpubr package and statistically analyzed by applying the non-parametric Wilcoxon signed-rank test on the values, where significance is set at *p*-value <0.05. Soluble molecule data were associated with other experimental data by Spearman correlation and visualized using the corrplot package.

### Immunohistochemistry (IHC), Immunofluorescence (IF), and GIEMSA staining

Slides (5 µm) from formalin-fixed paraffin embedded (FFPE) samples were processed for deparaffinization and rehydration. Heat-induced antigen retrieval (citrate buffer pH 6 or 9, Thermofisher) was performed using the microwave. 3% H_2_O_2_ incubation was used for IHC preparation. Slides were incubated ON at 4 °C with mouse anti-human CD66b antibody (BioLegend, 305102, 1:100 for both IHC and IF), anti-human CD8 (Invitrogen, 53-0008-82, 1:100), rabbit anti-human GZMK (Invitrogen, LS-C119554-50, 1:100), rabbit anti-human E-cadherin (Abcam, Ab1416, 1:100), rat anti-human Ki67 (eBioscience, SolA15, 1:200), rabbit anti-human EPCAM (Abcam, Ab32394, 1:400), mouse anti-human SDF1 (R&D, MAB350, 1:100), anti-human αSMA (Abcam, Ab8211, 1:200) in a blocking solution composed of 3% BSA, 5% goat serum (Invitrogen, 10000 C) and 0.1% Triton in PBS. After incubation with HRP-secondary antibody (30 min) and Aminoethyl Carbazole (AEC) + High Sensitivity Substrate Chromogen (Dako) (for IHC) or fluorophore-conjugated antibodies (Invitrogen, A32723, goat anti-mouse 488; Invitrogen, A21247, goat anti-rabbit 647; Invitrogen, A21206, goat anti-rabbit 488; Invitrogen, A21424, goat anti-mouse 555; 1:500; Invitrogen, A21434, goat anti-rat 555, all 1:500 for 1 h at RT), slides were mounted and acquired by using Aperio or SP8 confocal microscope (Leica), respectively. GIEMSA staining was performed on neutrophils freshly isolated from PB or sorted from tumors following manufacturing instruction (Hemacolor; Cat 1.11661.0001, Sigma).

### Imaging analysis

For quantification of GZMK signal on human colorectal cancers, sections were labeled with DAPI, anti-GZMK, anti-CD66b and Alexa Fluor 488-conjugated anti-CD8 primary antibodies and images captured with a Nikon CSU-W1 spinning disk (Nikon Europe BV) using a 40×/1.15 NA water immersion objective lens (pixel size 0.1625 × 0.1625 um^2^). For the tumor area identification, previews of tissue sections on the DAPI channel were acquired with a 10×/0.3 NA dry objective lens. Inside the tumor areas, 3 to 4 regions were randomly chosen for the acquisition with a 40×/1.15 NA water immersion objective lens. Each region was made of 12 adjacent tiles covering a total area >4 mm^2^ per tissue section.

The quantification of the GZMK signal in CD8^+^ cells was done with a custom-made Fiji macro^103^. Briefly, images were pre-processed with denoising and background subtraction, and nuclei were segmented on the DAPI channel with the plugin StarDist^104^, using the built-in model Versatile (fluorescent nuclei) and the intensity parameters, including “mean intensity” and “raw integrated density”, of CD8, GZMK and CD66b fluorescence were quantified in a band of thickness 1 μm around each nucleus.

Statistical analysis was performed in R. CD8^+^ cells were identified from the database by setting criteria on the intensity parameters of the specific fluorescence. Particularly, a cell was considered as positive for CD8 fluorescence channel if its “raw integrated density” was above a threshold value, set by the comparison in images of the “raw integrated density” values of cells expressing CD8 fluorescence and the “raw integrated density” of cells expressing only autofluorescence signal. Moreover, a further correction regarding the cells that had the band around the nucleus overlapping with red blood cells was applied: the ratio of the “raw integrated density” in the green channel versus the red channel was computed and only cells with a ratio green/red > 4 were considered as positive for CD8. The same procedure described above was used for the detection of CD66b positive cells (for red blood cells correction a ratio red/green > 2 was set to consider cells as CD66b positive).

Differences between two groups were calculated by unpaired, two-tailed Student’s t test. The analysis of GZMK fluorescence in CD8^+^ cells lying in the proximity of a neutrophil (CD66b^+^) was performed as follows: the distance between a CD8^+^ cell and its nearest neutrophil was computed per each field of view. Only distances below 10 μm were taken into account to select pairs of CD8^+^ cell-neutrophil that were close to each other. The mean intensity of GZMK fluorescence in CD8^+^ cells close to neutrophils was compared to the GZMK mean intensity of cells close to neutrophils that were not positive for CD8.

In order to evaluate the expression of GZMK of CD8^+^ cells in relation to their distance from a neutrophil, we performed the computation of lymphocytes and neutrophils distances in Tumor Area. The distances of CD8^+^ cells from each neutrophil were calculated in each acquired image using an R script and these distances were filtered with a min function in order to consider the «nearest» neutrophil per each CD8^+^ cell. For the analysis, we considered only distances <10 µm (Touching) and between 10 and 20 µm (Near). To account for the high variability in GZMK expression between patients, we normalized each cell’s intensity value of GZMK with respect to the Mean Intensity value of GZMK calculated for the Near category of the corresponding patient.

For the quantification of SDF-1 in cells positive for αSMA, human colorectal cancers sections labeled with DAPI, anti-SDF-1, anti-αSMA, and anti-EPCAM primary antibodies, and captured with a Nikon Eclipse Ti widefield microscope (Nikon Europe BV) using a 20×/0.75 NA dry objective lens (pixel size 0.32 × 0.32 µm^2^). For the tumor area’s identification, previews of tissue sections were acquired with a 4×/0.2 NA dry objective lens. Inside the tumor areas, regions were randomly chosen for the acquisition with 20×/0.75 NA dry objective lens for a total acquired area >35 mm^2^ per tissue section.

The quantification of SDF-1 signal in αSMA^+^ cells was done with Qupath v. 0.2.3^105^ through a custom-made script. Briefly, cell nuclei were detected on DAPI channel with the StarDist extension and a cell expansion of 1 um was applied in order to measure fluorescence in the cytoplasmic/membrane cell’s area. For the nuclei segmentation with StarDist, a custom model was trained and tested using the StarDist (2D) network in ZeroCostDL4Mic^106^: a training dataset was generated by the manual annotation of nuclei in QuPath and labeled images were exported for the deep-learning training; the model’s training and quality control assessment were performed with the StarDist (2D) notebook. For the detection of pixels positive for αSMA staining, a pixel classifier was created with the QuPath’s function Create thresholder and the quantification of the positive area inside each segmented cell was added as a measurement parameter in the detection’s results table. Other two-pixel classifiers in the red and far-red channels were similarly created to be used in the statistical analysis step. The expression of SDF1 was quantified as the mean intensity of the corresponding fluorescence signal in the band around the nucleus.

Statistical analysis was performed in R. Cells with an area > 3 µm^2^ (estimated as the 10% of the area of a band created around a nucleus with area size 46 µm^2^) of pixels classified as positive for αSMA were considered as αSMA^+^. Cells overlapping with red blood cells were discarded from the analysis when presenting positive area >0 µm^2^ for the pixel classifiers in green, red, and far-red channels. Differences between two groups were calculated by unpaired, two-tailed Student’s t test.

### scRNA-seq

T and NAT single cell suspension after counting was resuspended in PBS without Ca^2+^ and Mg^2+^ with 0.04% BSA. Approximately 2000 cells/µl from each sample were used for the analysis. Briefly, every sample was loaded into one channel of Single Cell Chip A using a Chromium Single Cell 3′ v2 Reagent Kit (10x Genomics). After capture and lysis, complementary DNA was synthesized and amplified over 14 cycles according to the manufacturer’s protocol (10x Genomics). Libraries were prepared from 50 ng amplified cDNA. Sequencing was performed using a NovaSeq 6000 System (Illumina). An average sequencing depth of at least 50,000 reads per cell was obtained for each sample.

### scRNAseq analysis

Single cells of samples from 11 patients (each one sequenced in the far tumor (D) and tumor site (T)) were sequenced with the 3’ v3 chemistry and aligned using Cellranger count v. 3.1 on human genome hg38. Only droplets with a minimum number of unique molecular identifiers (UMI) were considered as “cells”; this was done by default by the Cellranger program.

Based on total cells in each patient and the distribution of each patient’s cells within groups identified by UMAP, we excluded patients 3, 8, and 6 from further analyses. Thus, 52316 cells were processed.

We used the Seurat v. 3R package to analyze single cell data^107^. To identify cell populations or sub-populations, uniform manifold approximation and projection (UMAP) was carried out from the first principal components of the PCA based on the expression of the most variable genes, normalized using SCT transformation as suggested in Seurat documentation (“SCTransform” function^108^. The different clusters in UMAPs were found using nearest neighbor clustering (SNN) with the function “FindClusters” of the Seurat package. In particular:

- For global cell population (52316 cells, Supplementary Fig. 6A): for UMAP, the first 30 principal components were used, and n.neighbors=60, min.dist=0.005 were set. For SNN clustering, top 20 principal components were used, with other parameters k.param=10, prune.SNN = 1/10. The resolution used in the “FindClusters” function was set to 0.11.
- For T/NK cells (8716 cells, fig. Supplementary Fig. 6E): cells belonging to the “T/NK” cluster of the previous step (a) were used. The UMAP was built using the first 30 principal components and n.neighbors=40, min.dist=0.002 were set as parameters. SNN clustering was carried out as above (a) but with a resolution of 0.09 in the “FindClusters” function. Finally, cells with the first UMAP dimension (UMAP_1) > 8 were considered as outliers and removed.
- To further characterize T CD8^+^ cell subtypes (Fig. 6A), cells belonging to the clusters 3 and 4 were extracted from the previous step (b) and the UMAP was built using the first 15 principal components from the expression of the following cell-specific markers that presumably would identify the expected subtypes: *TRDC, PRF1, CD3D, CD3E, TRGC1, TRGC2, LEF1, IL6R, NOSIP, SELL, CCR7, CD4, BATF, TIGIT, FOXP3, TNFRSF4, CD8A, CD8B, GZMH, FGFBP2, GZMB, GNLY, ZEB2, HLA-DRB5, CX3CR1, GZMA, IFNG, CCL4, SBF2, KLRB1, LTB, IL7R, FOS, CD40LG, DPP4, IRF1, GZMM, GZMK, HLA-DRB1, HLA-DRA, CCL5, CTLA4, PDCD1, HAVCR2, LAG3, CD38, ENTPD1, LAYN, CXCL13,* and *TOX*. Other parameters for the UMAP were n.neighbors=20, min.dist=0.01. For SNN clustering, the top 15 principal components were used, with other parameters being k.param=10, prune.SNN = 1/10. The resolution parameter in the “FindClusters” function was set to 0.5.

Differential expression of genes between different cell subgroups was carried out with the “FindMarkers” function in the Seurat package, using the Wilcoxon test. Thresholds were set to log2FC = 0.25 and adjusted *p* value = 0.05. For Fig. 6C, F and Supplementary Fig. 7D, E the size of the points corresponds to the score of the specific signature; this score is defined, for each cell, as the sum of the counts of the genes belonging to a specific signature/the total sum of the counts of that cell. The points with the biggest size correspond to a score higher than 80% of the distribution of the scores of all the cells.

Cell types in the entire population were identified with the help of scMCA package^109^ and validated against the respective human cell-type markers. Pseudotime analysis was done using Monocle v. 3.

### In vivo animal experiments

C57BL/6J were subcutaneously injected with 1 × 10^6^ MC38 cells/mouse. Animals were euthanized when the tumor reached the volume of <1 cm^3^ and >1 cm^3^ to evaluate CD8^+^ T cell and neutrophil infiltration during progression. Animal were euthanized when tumors become ulcerated or interfere with the ability of the animal to eat, drink, or ambulate. Blood was collected and spleen and tumors harvested and dissociated to a single cell suspension. Tumors were digested (1.5 mg/mL collagenase, 0.75 mg/mL hyaluronidase, and 0.1 mg/mL DNase I) in RPMI 10% FBS at 37 °C. Cell suspension was filtered through 70 μm cell strainers, washed, counted, and stained for multiparametric flow cytometry. The depletion of neutrophils in vivo was obtained by intraperitoneal injection of anti-Ly6G (clone 1A8, #BP0075-1, Bio X Cell) and anti-rat mouse IgG2a, Kappa immunoglobulin (clone MAR 18.5, #BE0122, Bio X Cell) or isotype controls (#BP0089 for anti-Ly6G and #BE0085 for anti-rat mouse IgG2a)^110^ based on the following treatment schedule: (1) anti-Ly6G or isotype: daily (day 0-20), i.p., 25 μg/mouse for day 0–6, and 50 μg/mouse for day 7–18; (2) anti-rat mouse IgG2a or isotype: every other day (day 0/2/4/6/8/10/12/14/16/18/20), i.p., 50 μg/mouse, i.p.; (3) for the combo injection days, injection of anti-rat IgG2a first, and 2 h later, do the injection of anti-Ly6G. Mice were euthanized for analysis at day 20. All mouse experiments were conducted in agreement with requirements permitted by our ethical committee.

### Survival analysis methods

We investigated the interplay between immune cytolytic activity and neutrophils infiltration on overall (OS) and disease-free survival (DFS) in the colon adenocarcinoma cohort (COAD) of The Cancer Genome Atlas (TCGA)^111^ by performing a Kaplan–Meier analysis. The cytolytic activity index was quantified by computing the geometric mean of granzyme A and perforin expression^112^ and samples were split according to the Maximally Selected Ranks Statistics cutoff (“maxstat”, version 0.7-25). Neutrophils relative abundance was obtained by running CIBERSORTx^112^ with a validated leukocyte gene signature matrix (LM22) on RNAseq data of COAD samples. Samples were divided into two groups according to the presence or absence of Neutrophils. Similarly, CIBERSORTx-derived gene expression signature was employed to infer the GZMK^high^ cell subtype’ abundance and analyze its association with survival. First, the single-cell raw counts matrix of CD8^+^T_EM_ population was split into two subgroups expressing “high” or “low” values of GZMK and used it to feed CIBERSORTx, building a gene signature able to discriminate the two cell sub-populations. Secondly, we collected survival tables of TCGA-COAD and TCGA-LUAD cohorts from cBioPortal^113^ and ran Kaplan-Meier analysis stratifying patients with “high” and “low” infiltration of CD8^+^ T_EM_ cells expressing high levels of GZMK. Survival analyses were carried out on R software version 4.0.5), by using “survival” (version 3-2-11) and “survminer” (version 0.4.9) packages.

### Statistics and data visualization

Results were analyzed and visualized on Prism version 9.2.0 (GraphPad). All statistical analyses were conducted using GraphPad Prism version 9.2.0 or R software version 4.0.2. Differences between two groups were calculated by two-tailed Student’s t test, unless otherwise specified. For graphs with multiple comparisons being made, one-way ANOVA was performed with post hoc Sidak’s test or Tukey’s test for multiple comparisons, unless otherwise specified. Significance was set at *p* values ≤ 0.05. For all figures: *, *p* ≤ 0.05; **, *p* ≤ 0.01; ***, *p* ≤ 0.001. All *p* values are indicated in the figure legend. All plots indicate Min to Max value data and error bars are shown as mean ± SEM. The *n* numbers for each experiment and the numbers of experiments are annotated in each figure legends. For all experiments, data are reported based on individual biological replicates pooled from multiple donors and animals.

### Reporting summary

Further information on research design is available in the Nature Portfolio Reporting Summary linked to this article.

## Supplementary information

Supplementary Information

Reporting Summary

## Data Availability

The transcriptomic data generated in this study have been deposited in the European Genome-Phenome Archive (EGA) under accession code Study: EGAS00001006665-Dataset: EGAD00001009634. The transcriptomic data are available under restricted access in accordance with art. 13 General Regulation on Data Protection (EU Regulation 2016/679), access can be obtained by request to EGA and approval by the competent Data Access Committee. Please contact the corresponding authors for access requests. The TCGA-COAD and TCGA-LUAD gene expression data used in this study are publicly available in the GDC Data Portal (https://portal.gdc.cancer.gov/). Survival tables of TCGA-COAD and TCGA-LUAD cohorts were downloaded from cBioPortal (https://www.cbioportal.org/). The remaining data are available within the Article, Supplementary Information or Source Data file. Source data are provided with this paper.

## Source data

Source Data

## Code availability

Code to reproduce Kaplan–Meier plots of the manuscript can be found here (https://github.com/DanCag/survival-analysis). The remaining data are available within the Article, Supplementary Information or Source Data file.
