# Author Correction: GZMK^high^ CD8^+^ T effector memory cells are associated with CD15^high^ neutrophil abundance in non-metastatic colorectal tumors and predict poor clinical outcome

**DOI:** 10.1038/s41467-022-35251-z

**Published:** 2022-11-30

**Authors:** Silvia Tiberti, Carlotta Catozzi, Ottavio Croci, Mattia Ballerini, Danilo Cagnina, Chiara Soriani, Caterina Scirgolea, Zheng Gong, Jiatai He, Angeli D. Macandog, Amir Nabinejad, Carina B. Nava Lauson, Arianna Quinte’, Giovanni Bertalot, Wanda L. Petz, Simona P. Ravenda, Valerio Licursi, Paola Paci, Marco Rasponi, Luca Rotta, Nicola Fazio, Guangwen Ren, Uberto Fumagalli-Romario, Martin H. Schaefer, Stefano Campaner, Enrico Lugli, Luigi Nezi, Teresa Manzo

**Affiliations:** 1 Department of Experimental Oncology, European Institute of Oncology- IRCCS, Milano, Italy; 2 Center for Genomic Science of IIT, CGS@SEMM (Istituto Italiano di Tecnologia at European School of Molecular Medicine), Fondazione Istituto Italiano di Tecnologia (IIT), Milano, Italy; 3 Laboratory of Translational Immunology, IRCCS- Humanitas Research Hospital-, Milan, Italy; 4 The Jackson Laboratory, Bar Harbor, ME USA; 5 Department of Molecular & Biomedical Sciences, University of Maine, Orono, ME 04469 USA; 6 Department of Pathology, APSS Trento Hospital & Health Care, Trento, Italy; 7 Digestive Surgery Division, European Institute of Oncology-IRCCS, Milan, Italy; 8 Division of Gastrointestinal Medical Oncology and Neuroendocrine Tumors, European Institute of Oncology-IRCCS, Milan, Italy; 9 Institute of Molecular Biology and Pathology (IBPM), National Research Council (CNR) of Italy, Rome, Italy; 10 Department of Computer, Control and Management Engineering, Sapienza - University of Rome, Rome, Italy; 11 Department of Electronics, Information and Bioengineering, Politecnico di Milano, Milan, Italy

**Keywords:** Tumour immunology, Colorectal cancer

Correction to: *Nature Communications* 10.1038/s41467-022-34467-3, published online 08 November 2022

In this article the funding from the Italian Ministry of Health with Ricerca Corrente and 5x1000 was omitted. The original article has been corrected.

