# Supplementary material for: GZMKhigh CD8+ T effector memory cells are associated with CD15high neutrophil abundance in non-metastatic colorectal tumors and predict poor clinical outcome
Source: Nat Commun. 2022 Nov 8;13:6752. doi: 10.1038/s41467-022-34467-3 (PMC9643357; doi:10.1038/s41467-022-34467-3)
Supplement: Supplementary file 1 — Supplementary Information [file 41467_2022_34467_MOESM1_ESM.pdf]

SUPPLEMENTARY INFORMATION.

GZMK^{high} CD8^{+} T effector memory cells are associated with CD15^{high} neutrophil abundance in non-metastatic colorectal tumors and predict poor clinical outcome.

Silvia Tiberti[1], Carlotta Catozzi[1], Ottavio Croci[2], Mattia Ballerini[1], Danilo Cagnina[1], Chiara Soriani[1], Caterina Scirgolea[3], Zheng Gong[4], Jiatai He[4,5], Angeli D. Macandog[1], Amir Nabinejad[1], Carina B. Nava Lauson[1], Arianna Quinte'[1], Giovanni Bertalot[6], Wanda L. Petz[7], Simona P. Ravenda[8], Valerio Licursi[9], Paola Paci[10], Marco Rasponi[11], Luca Rotta[1], Nicola Fazio[7], Guangwen Ren[4], Uberto Fumagalli-Romario[7], Martin H. Schaefer[1], Stefano Campaner[2], Enrico Lugli[3], Luigi Nezi[1*] and Teresa Manzo[1*].

* These authors jointly supervised this work

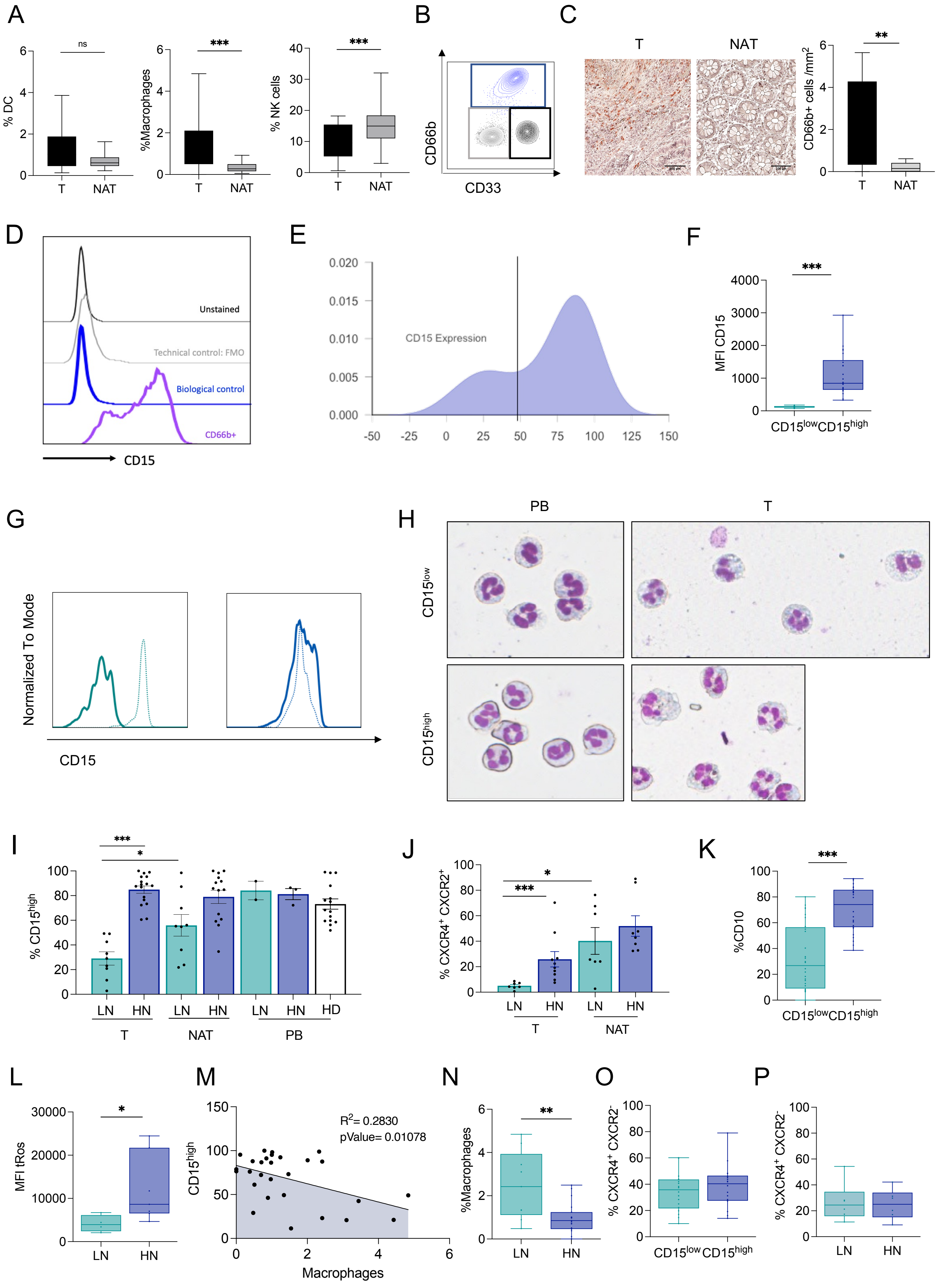

Supplementary Figure 1

Supplementary Figure 1. CD15^{high} neutrophils in CRC patients.

A. Dendritic cells (DC) (n=20, p=0.162), macrophages (n=27, p<0.001) and NK cells (n=22, p<0.001) within CD45$^+$ cells in Tumor (T) and normal adjacent tissue (NAT) **B.** Representative contour plot of neutrophils (CD11b$^+$/HLA-DR-/CD66b$^+$, blue), Monocytic-Myeloid Derived Suppressor Cells (M-MDSCs) (CD11b$^+$/HLA-DR$^-$/CD66b$^-$/CD33$^+$, black), others myeloid components (grey). **C.** Representative immunohistochemistry images and CD66b$^+$ quantification in paired T and NAT (n=13, p=0.0057). Scale bar 100µm. **D.** CD15 staining in CD66b$^+$ (purple) and in CD66b$^-$ (blue), FMO for CD15 (grey) and unstained control (black). **E.** Density plot of CD15^{high} neutrophils in CRC cohort (n=27). Black line represents the anti-mode of the distribution. **F.** CD15 MFI within CD15^{low} and CD15^{high} neutrophils (n=22, p<0.001). **G.** Representative histogram of CD15 expression on neutrophils in T and NAT within LN and HN patients. **H.** GIEMSA staining of CD15^{low} and CD15^{high} neutrophils sorted from PB and T. **I.** CD15^{high} neutrophils quantification within T, NAT, and PB of LN (n=9) and HN (n=17) patients and PB of healthy donors (HD) (n=15) (p=0.043 for LN-NAT vs LN-T, p<0.001 for HN-T vs LN-T). **J.** CXCR4$^+$ CXCR2$^+$ neutrophils within T and NAT of LN (n=7) and HN (n=10) patients (p=0.016 for LN-NAT vs LN-T, p< 0.001 for HN-T vs LN-T). **K.** Frequency of CD10$^+$ within CD15^{low} and CD15^{high} neutrophils (n=28, p< 0.001). **L.** Total ROS (tRos) MFI within neutrophils of LN and HN patients' tumor (n=11, p=0.024). **M.** Pearson correlation between CD15^{high} neutrophils and macrophages (n=28, p=0.0107) **N.** Frequency of macrophages within LN (n=9) and HN (n=19, p=0.001). **O.** Frequency of CXCR4$^+$CXCR2$^-$ within CD15^{low} and CD15^{high} neutrophils (n=18, p=0.299). **P.** Frequency of CXCR4$^+$CXCR2$^-$ within LN (n=6) and HN (n=6, p=0.826) patients. The error bars represent mean ± SEM or box and whisker plots indicate Min to Max value; two-tailed unpaired t-test (A,I,J,N,P), two-tailed paired t-test (C,F,I,J,K), two-tailed Mann-Whitney test (L), two-tailed Wilcoxon-test (O). Source data are provided as a Source Data file.

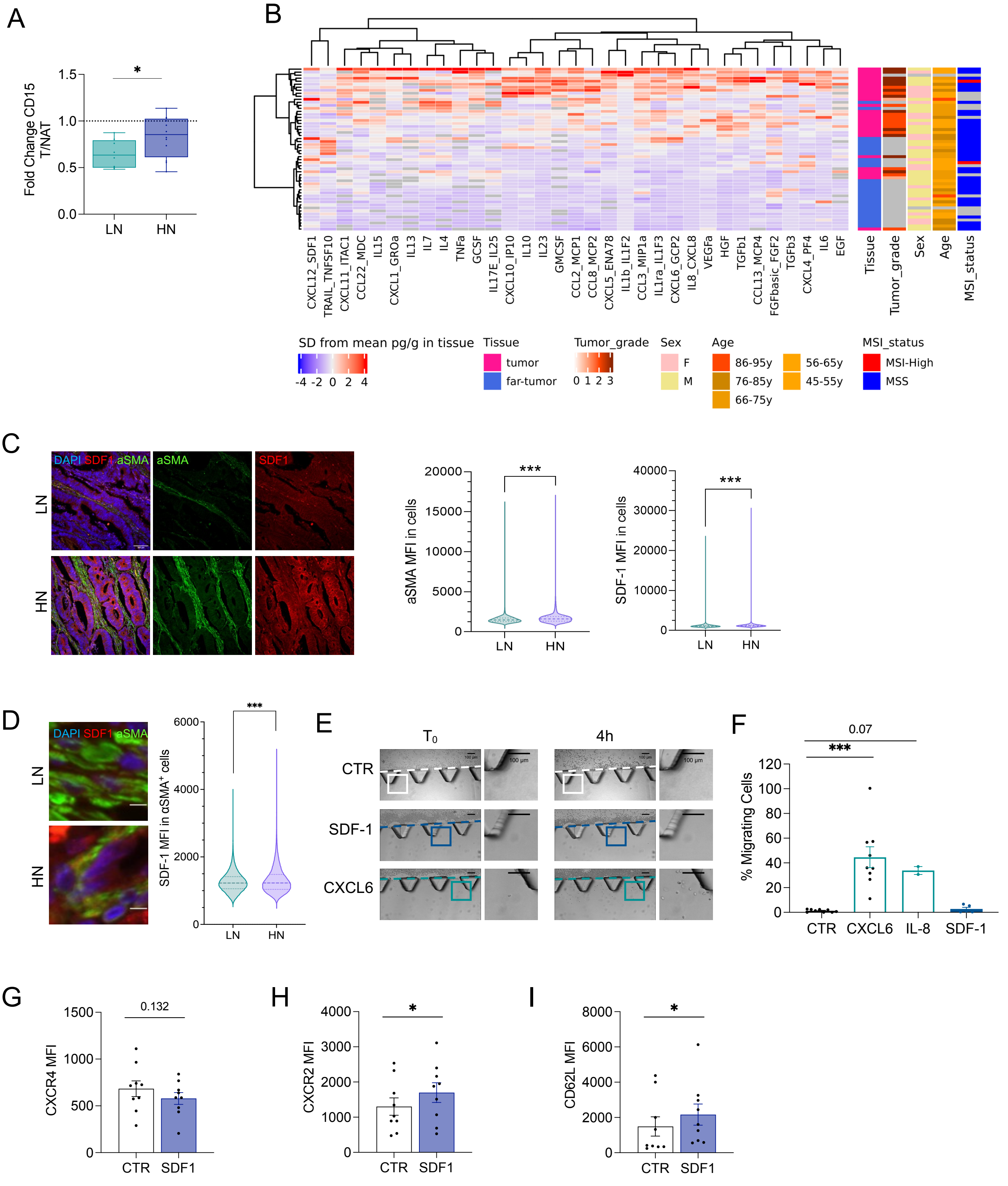

Supplementary Figure 2

Supplementary Figure 2. Stromal Derived Factor-1 (SDF-1) promotes neutrophil's retention within CRC tumors.

A. Fold change of CD15 expression within neutrophils from HD treated with interstitial fluid (IF) from LN (n=8) or HN (n=12, *p*=0.0537) patients. **B.** Heatmap showing the abundance of soluble molecules derived from IF of T and NAT (n=54). Soluble molecules clustered by Ward's minimum variance method, whereas samples by Euclidean distance. Tissue type, tumor grade, sex, age and Microsatellite Instability (MSI) status are indicated in the figure. **C- D.** Representative images and quantification by confocal microscopy of SDF-1 and aSMA (C, *p*<0.001) and SDF-1 in aSMA$^+$ cells (D, *p*<0.001) on FFPE tumor tissues from LN (n=4) and HN (n=5) patients. DAPI blue, aSMA green, SDF-1 red. Scale bar 100µm. **E- F.** Representative images (E) and quantification (F) of migrating neutrophils on chip upon stimulation with CXCL6 (400 ng/mL) (n=10), IL-8 (100 ng/ml) (n=2), SDF-1 (100 ng/ml) (n=4) or culture medium (CTR, n=8); *p*<0.001 for CXCL6 *vs* SDF-1 and CXCL6 *vs* CTR, *p*=0.82 for CXCL6 *vs* IL8, *p*=0.99 for SDF-1 *vs* CTR, *p*=0.12 for SDF1 *vs* IL8, *p*=0.07 for CTR *vs* IL8). Scale bar 100µm. **G-I.** CXCR4 (G; *p*=0.132), CXCR2 (H; *p*=0.020) and CD62L (I; *p*=0.039) MFI in neutrophils isolated from HD treated with SDF-1 culture medium (CTR, n=9). Bars represent mean ± SEM or box and whisker plots indicate Min to Max value. Two-tailed unpaired t test (A,C), two-tailed one-way Anova (F). Source data are provided as a Source Data file.

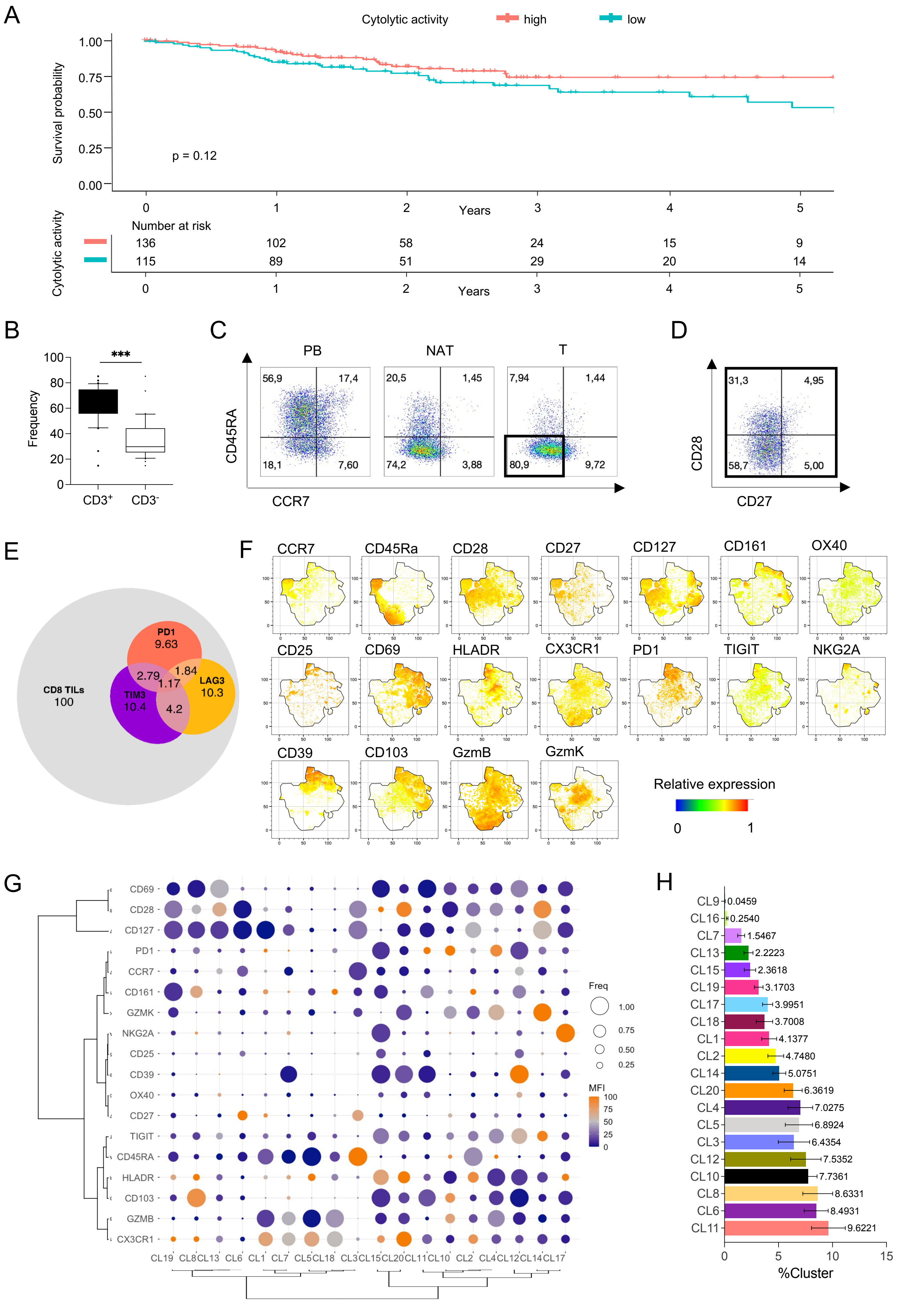

Supplementary Figure 3

Supplementary Figure 3. CD8+ T cell landscape in CRC patients.

A. Kaplan-Meier analysis of the association of cytolytic activity (high in red, low in turquoise) signature with disease free survival (DFS) on the TCGA-COAD cohort (n=284; see Methods and Supplementary Table 1 for details). The table shows the 'number at risk' subjects. **B.** Frequency of $CD3^+$ and $CD3^-$ cells within $CD45^+$ cells in T (n=32, *p*=0.0001). **C.** Representative dot plot of CD45RA and CCR7 expression within $CD8^+$ T cells in PB, NAT and T. **D.** Representative dot plot of CD28 and CD27 expression within $CD8^+$ T_{EM}. **E.** Eulero Venn diagram of indicated markers' frequencies within $CD8^+$ T cells (n=34). **F.** Expression level of selected markers in the $CD8^+$ T cells. tSNE representation of concatenated PB, NAT and T samples. **G.** Balloon plot of the average expression levels and frequencies of 18 selected markers in the 18 clusters analyzed (n=34). **H.** Mean frequency of the 20 clusters of $CD8^+$ T cells identified by the unsupervised clustering analysis (n=34). Bars represent mean ± SEM or box and whisker plots indicate Min to Max value; two-tailed paired t test. Source data are provided as a Source Data file.

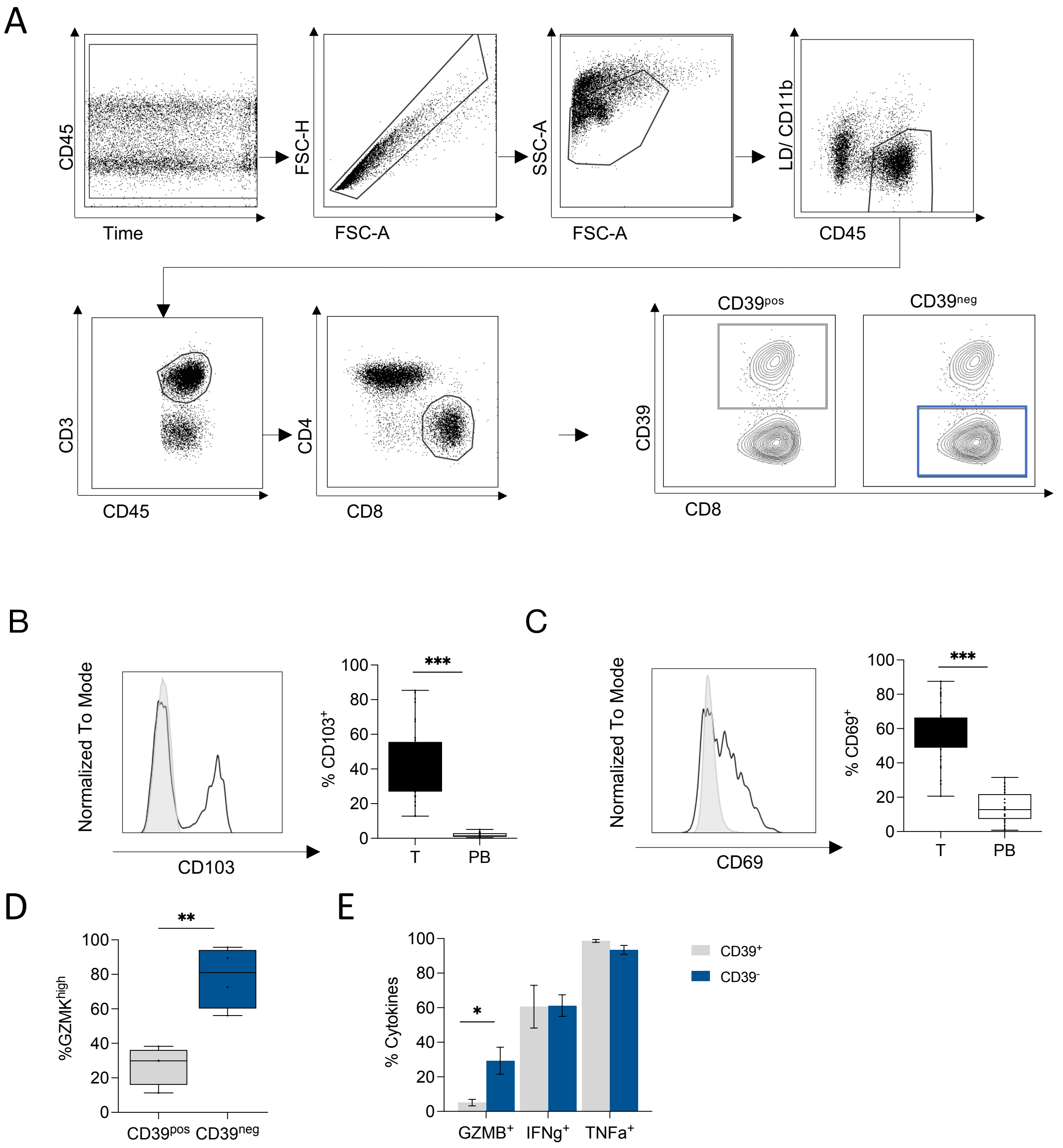

Supplementary Figure 4

Supplementary Figure 4. Characterization of CD39^{neg} GZMK^{high} population in CRC patients.

A. Flow cytometric gating strategy for the identification of CD39^{neg} GZMK^{high} population in CRC patients. **B-C.** Representative histogram and quantification of CD103 (B; *p*<0.001) and CD69 (C; *p*<0.001) frequency within CD39^{neg} CD8^{+} T_{EM} in T (n=34) and PB (n=18). **D.** GZMK^{high} frequency within sorted CD39^{pos} and CD39^{neg} CD8^{+} T_{EM} from T tissue (n=4, *p*=0.009). **E.** Frequency of GZMb (*p*=0.0328), IFNg^{+} (*p*=0.9667) and TNFa^{+} (*p*=0.1797) cells within sorted CD39^{pos} and CD39^{neg} CD8^{+} T_{EM} from T (n=4). Bars represent mean ± SEM or box and whisker plots indicate Min to Max value, two-tailed Wilcoxon test (A), two-tailed paired t test (B,C,D, E). Source data are provided as a Source Data file.

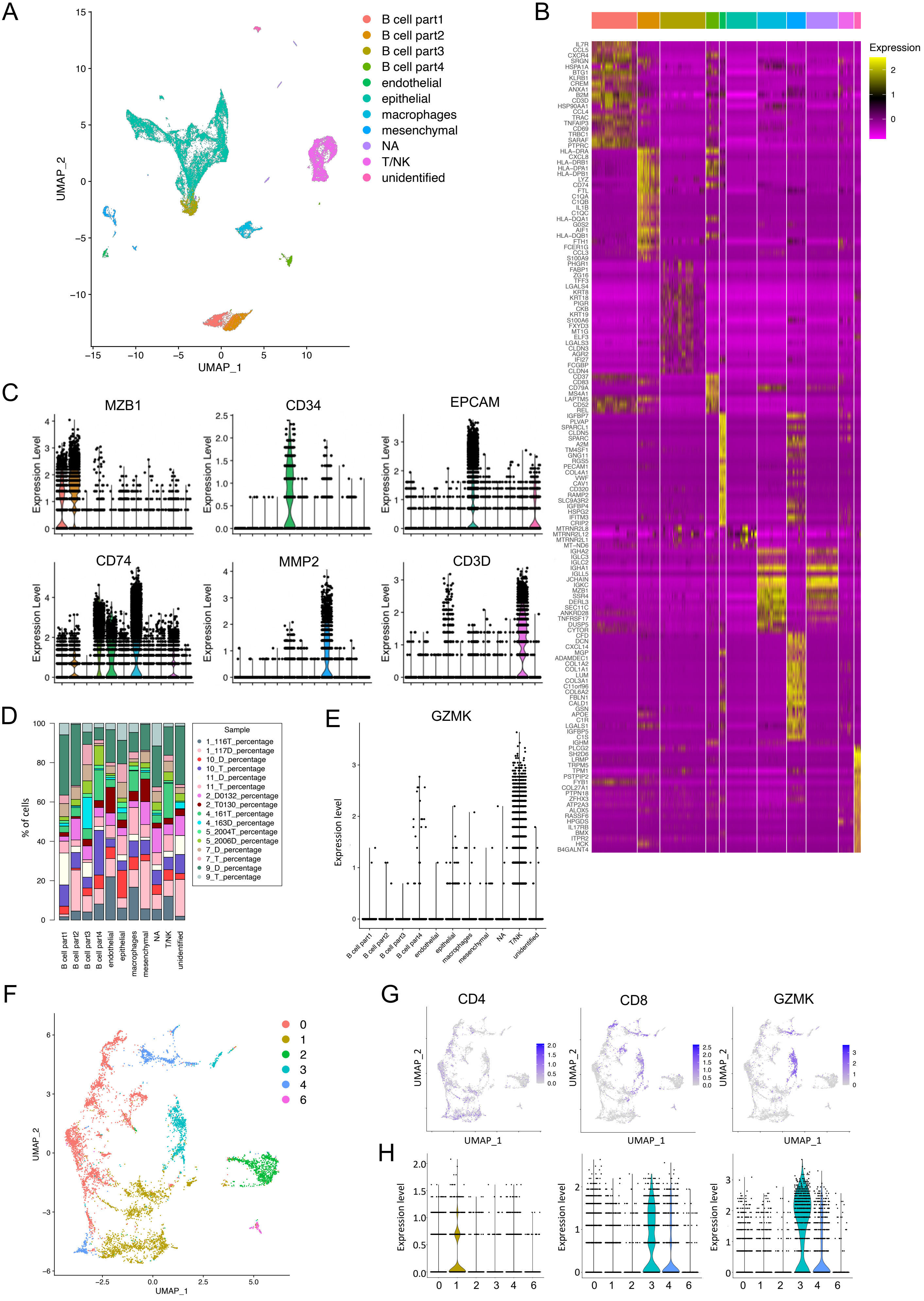

Supplementary Figure 5

Supplementary Figure 5. CD39^{neg} GZMK^{high} signature identified by RNAseq analysis.

A. UMAP projection of cells from 8 patients. Cells were colored based on the SNN-clustering. Each cluster was assigned to a cell type, as indicated (see Methods). **B.** Heatmap showing the scaled UMI counts of the differentially expressed genes found in each cluster. **C.** Violin plots showing normalized expression of selected cell type markers in the SNN cluster shown in (A). **D.** Bar plot showing patients' distribution of each cell cluster. Colors match the indicated tissue of origin. **E.** Violin plot showing the expression of *GZMK* in the indicated clusters from (A). **F-G.** UMAP projection of cells from the T/NK cluster in (A). Colors represent the SNN clusters (F). The expression of indicated T cell markers is shown (G). **H.** Violin plots showing the expression of selected genes for each cluster in T/NK cells.

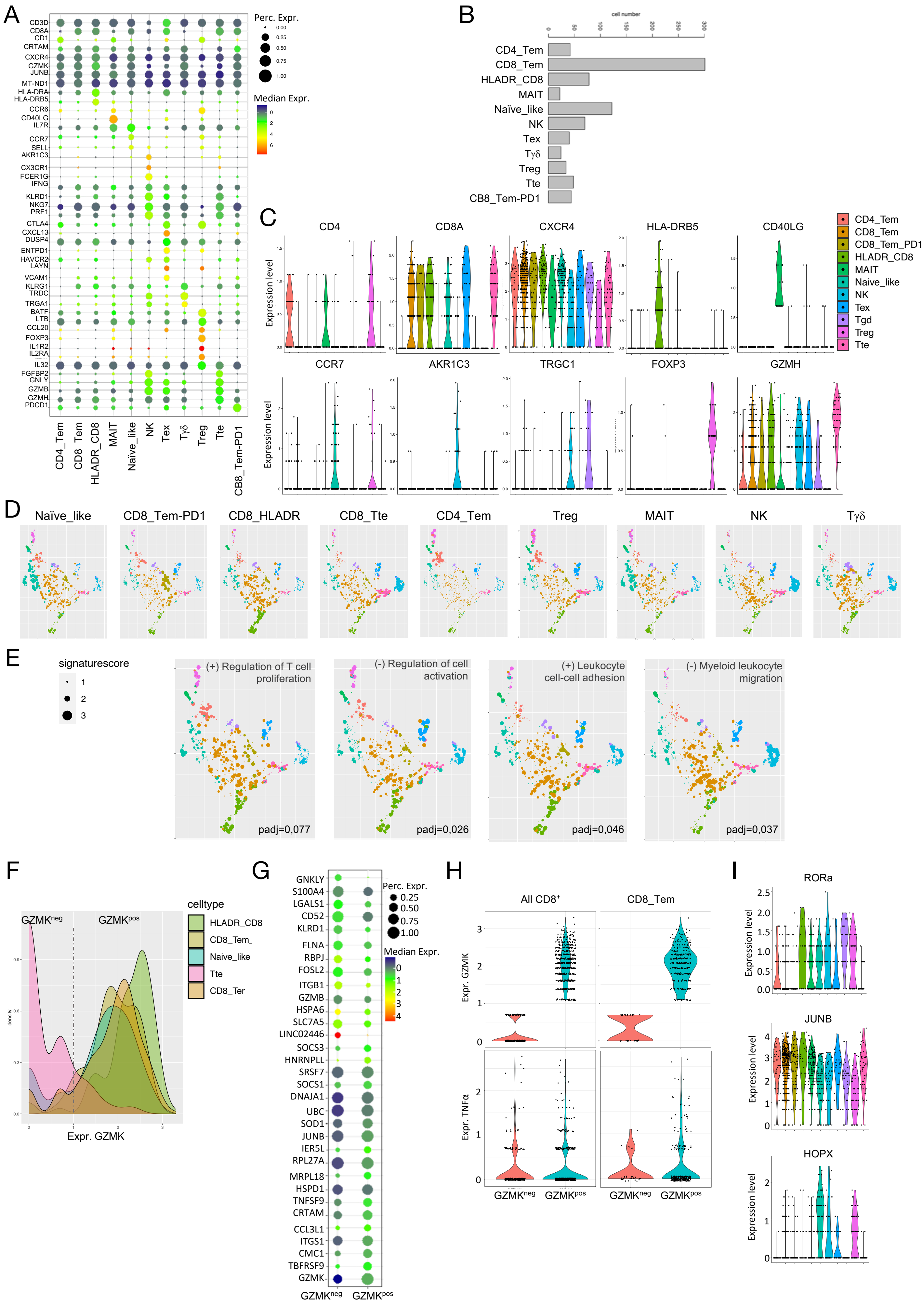

Supplementary Figure 6

Supplementary Figure 6. CD39^{neg} GZMK^{high} signature identified by RNAseq analysis.

A. Balloon plot showing the expression of selected markers for each manually-annotated T cell subtype (n= 8; see Supplementary Table 1 for list of genes used in the annotation). The size of the bubble represents the fraction of cells with at least one UMI for a specific gene, while the color shows the median of the scaled normalized expression in that fraction of cells. **B.** Bar plot showing the number of cells in each CD8^{+} T cell subtype (n= 8). **C.** Violin plots showing the expression of select marker genes in each of the indicated T cell subtypes (n= 8). **D-E.** UMAP showing the expression of the (D) indicated cell type signatures (see Supplementary . Table 1) or (E) immune signature from Reactome. Cells are color coded based on SNN-clustering; dot size reflects the expression of the signature in each cell (n= 8). **F.** Distribution plot showing *GZMK* expression in the indicated CD8^{+} T cell subtypes (n= 8). **G.** Balloon plot showing the expression of differentially expressed genes between GZMK^{pos} and GZMK^{neg} CD8_Tem cells. The size of the bubble represents the fraction of cells expressing a specific gene (n), while the color shows the median of scaled normalized expression of that fraction of cells. The threshold for considering a cell as GZMK^{pos} was set to UMI ≥1 (n= 8). **H**. Violin plots showing the expression of *GZMK* and *TNFa* in the entire CD8^{+} T cell population (left) and in CD8^{+} Tem (right) cells. Cells were considered positive for *GZMK* if the number of GZMK UMI was ≥1 (n= 8). **I.** Violin plots showing the expression of select genes in each of the indicated T cell subtypes, color coded as in (C) (n= 8).

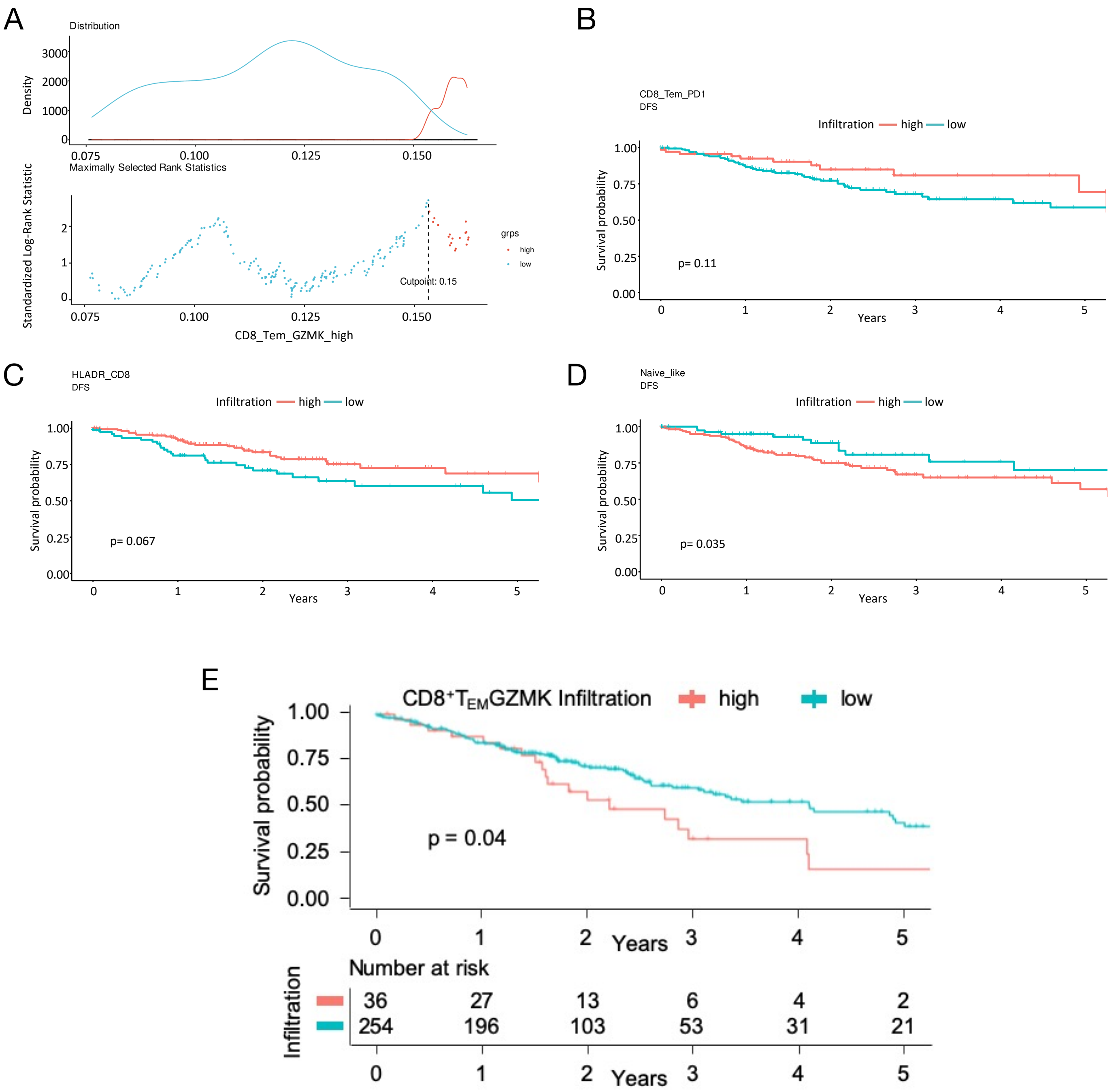

Supplementary Figure 7

Supplementary Figure 7. Prognostic value of CD39^{neg} GZMK^{high} signature identified by RNAseq analysis. **A.** Distribution of CD8_Tem cell subtype' infiltration across TCGA-COAD samples. The optimal cutpoint for stratifying high- and low-level infiltration was obtained by using the Maximally Selected Rank Statistics (n= 284). **B-D.** Kaplan-Meier analysis of the association of CD8_Tem_PD1 (B), HLADR_CD (C) and Naïve-like (D) T cell subtype' abundance with disease free survival (DFS) on the TCGA-COAD cohort (n= 284; see Methods and Supplementary Table 1 for details). The optimal cutpoint for stratifying high- and low-level infiltration was obtained by using the Maximally Selected Rank Statistics. **E.** Kaplan-Meier analysis of the association of CD8_Tem cell subtype' abundance with overall survival (OS) on the TCGA-LUAD cohort (n= 295; see Methods and Supplementary Table 1 for details). The table shows the 'number at risk' subjects.

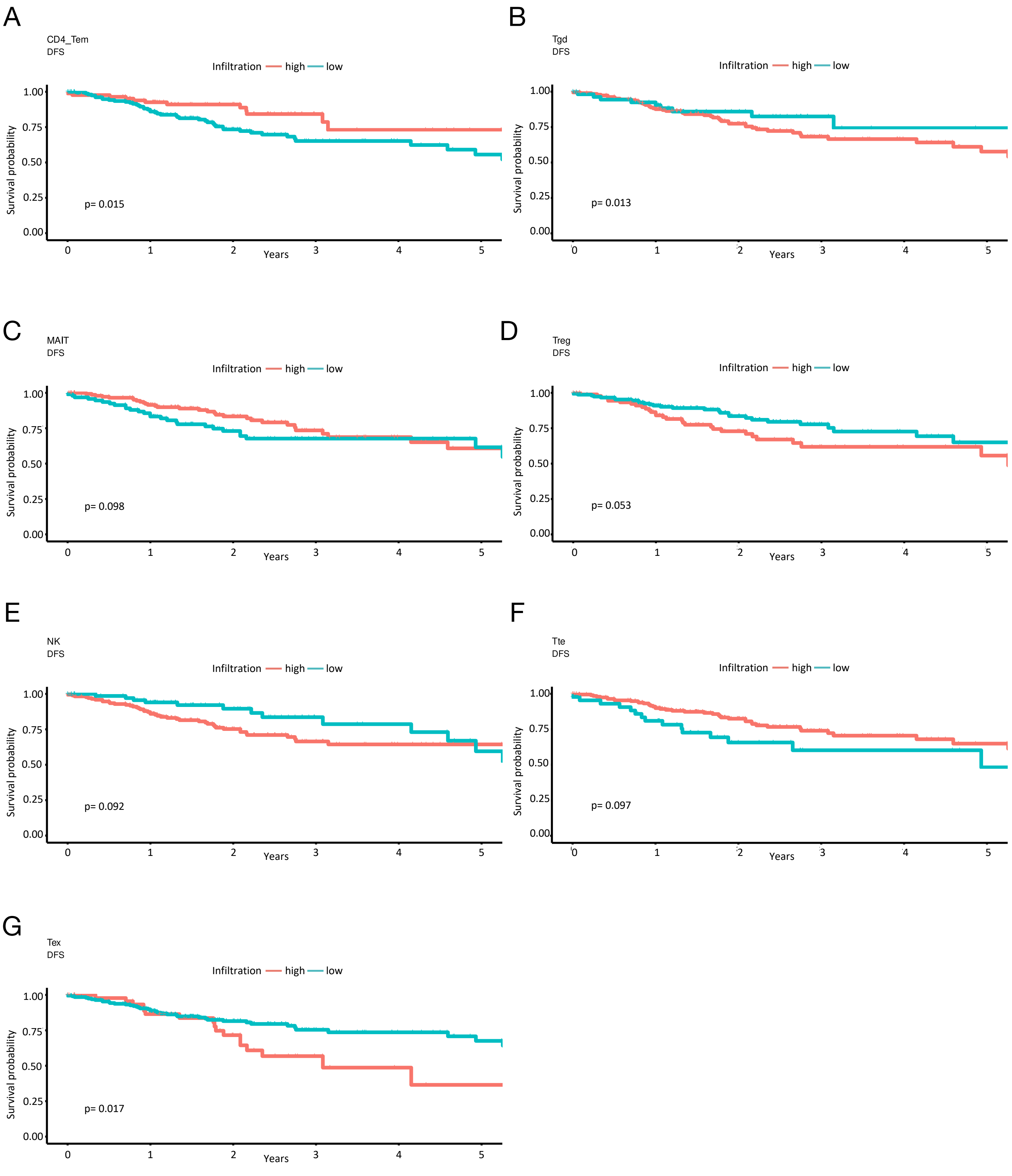

Supplementary Figure 8

Supplementary Figure 8. Prognostic value of CD39^{neg} GZMK^{high} signature identified by RNAseq analysis. Kaplan-Meier analysis of the association of CD4_Tem (**A**), Tgd (**B**), MAIT (**C**), Treg (**D**), NK (**E**), Tte (**F**) and Tex (**G**) T cell subtype' abundance with disease free survival (DFS) on the TCGA-COAD cohort (n= 284; see Methods and Supplementary Table 1 for details). The optimal cutpoint for stratifying high- and low-level infiltration was obtained by using the Maximally Selected Rank Statistics.

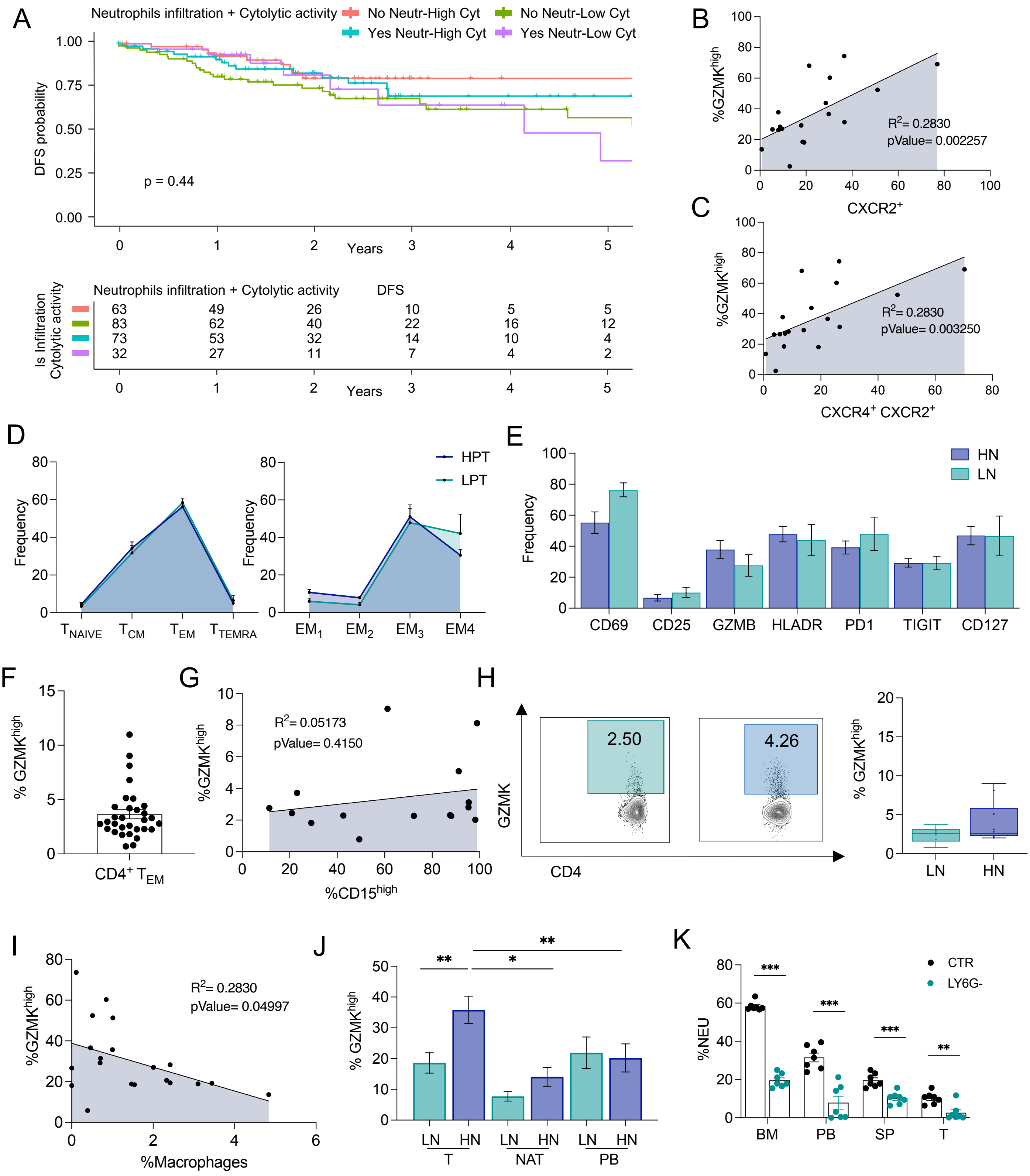

Supplementary Figure 9

Supplementary Figure 9. Specific interaction of CD15^{high} neutrophils and GZMK^{high} CD8^{+} T$_{EM}$ cells.

A. Kaplan-Meier of the association of cytolytic activity's and neutrophil's signature on disease free survival (DFS) on the TCGA-COAD cohort (n= 284; no neutrophils infiltration + high cytolytic activity in red, no neutrophils infiltration + low cytolytic activity in green, neutrophils infiltration + high cytolytic activity in turquoise, neutrophils infiltration + low cytolytic activity in violet). The 'number at risk' table is shown below the survival curves. **B.** Pearson correlation between CXCR2^{+} neutrophils and GZMK^{high} CD8^{+} T$_{EM}$ cells (n=20; *p*=0.0022). **C.** Pearson correlation between CXCR4^{+} CXCR2^{+} neutrophils and GZMK^{high} CD8^{+} T$_{EM}$ cells (n=20; *p*=0.0032). **D.** Frequency of Naïve (T$_{NAIVE}$), Central Memory (T$_{CM}$), Effector Memory (T$_{EM}$) and Effector Memory CD45RA^{+} (T$_{EMRA}$) T cells in LN (n=6) and HN (n=10) patients. Frequency of T$_{EM1-4}$ in LN (n=6) and HN (n=10) patients (*p*=0.9985 for T$_{NAIVE}$, *p*=0.9536 for T$_{CM}$, *p*=0.9745 for T$_{EM}$, *p*=0.9940 for T$_{EMRA}$, *p*=0.9153 for EM$_{1}$, *p*=0.9652 for EM$_{2}$, *p*=0.9808 for EM$_{3}$, *p*=0.2740 for EM$_{4}$. **E.** Expression level of the indicated markers within CD8^{+} T$_{EM}$ cells in LN (n=6) and HN (n=10) patients (*p*=0.0630 for CD69^{+}, *p*=0.3663 for CD25^{+}, *p*=0.3085 for GZMB^{+}, *p*=0.7048 for HLADR^{+}, *p*=0.3735 for PD1^{+}, *p*=0.9643 for TIGIT^{+}, *p*=0.9840 for CD127^{+}). **F.** Frequency of GZMK^{high} cells within CD4^{+} T$_{EM}$. **G.** Pearson correlation between CD15^{high} neutrophils and GZMK^{high} CD4^{+} T$_{EM}$ (n=15; *p*=0.4150). **H.** Contour plot and quantification in LN (n=6) and HN (n=10) patients (*p*=0.199). **I.** Pearson correlation between macrophages and GZMK^{high} CD8^{+} T$_{EM}$ cells (n=21; *p*=0.0499). **J.** Quantification of GZMK^{high} CD8^{+} T$_{EM}$ cells within LN (n=7) and HN (n=8) patients in T, NAT and PB (*p*=0.009 for HN-T vs LN-T, *p*=0.046 for HN-NAT vs HN-T, *p*=0.001 for HN-PB vs HN-T). **K.** Frequency of neutrophils in tumor (T), bone-marrow (BM), Spleen (SP) and blood (PB) of MC38 tumor bearing mice upon αLy6G antibody-mediated neutrophil's depletion or treatment with isotype control (*p*<0.0001 for BM and PB, *p*=0.0002 SP, *p*= 0.002 for T. Bars represent mean ± SEM or box and whisker plots indicate Min to Max value; two-tailed two-way Anova (D), two-tailed unpaired t test (E,H,I,K), two-tailed paired t test (I). Source data are provided as a Source Data file.

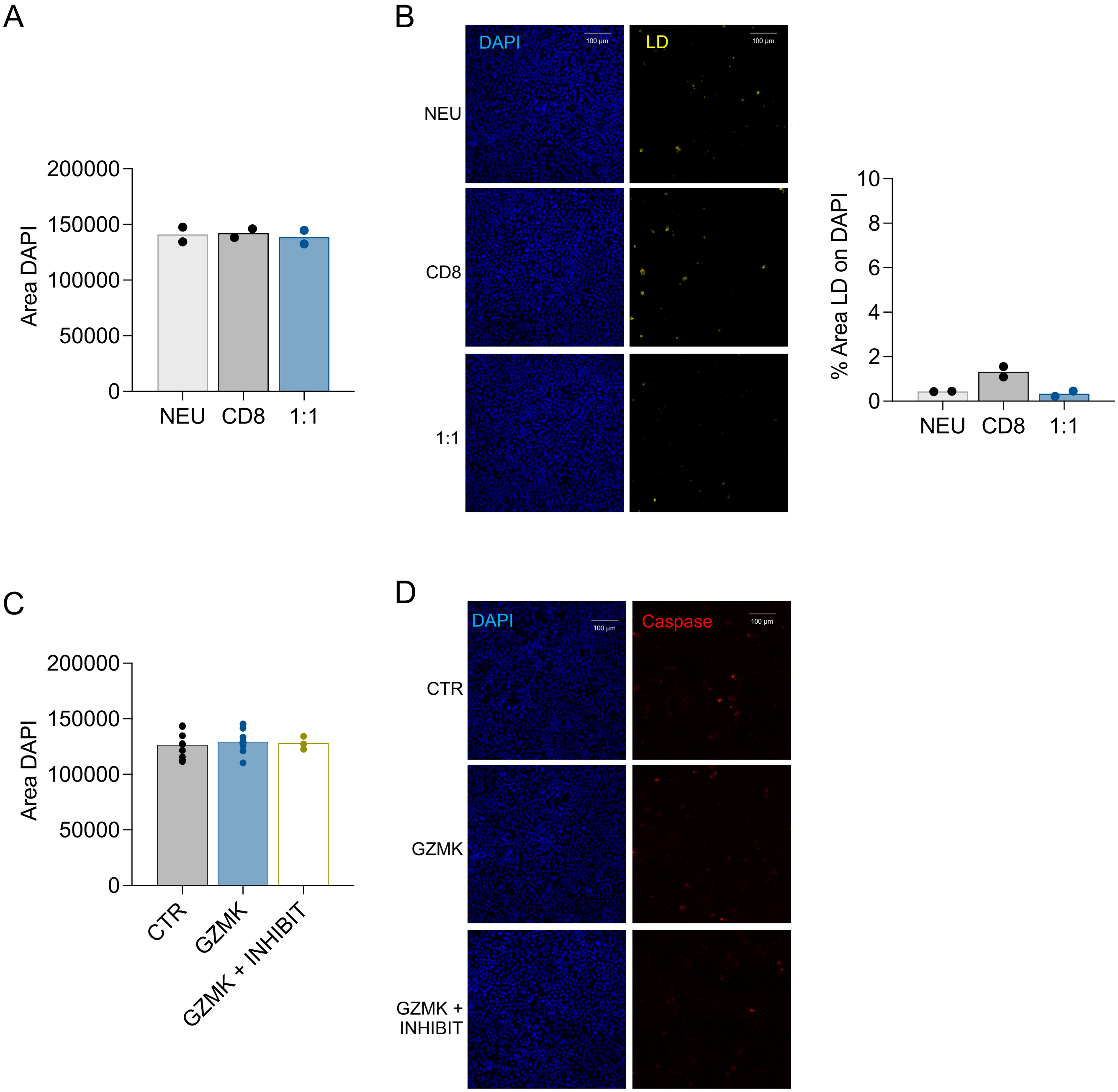

Supplementary Figure 10

Supplementary Figure 10. Granzyme k does not have a pro-apoptotic effect.

A-B. Representative images and quantification of DAPI positive area (A) and live-dead staining (B, LD) by multicolor confocal imaging on CACO-HT29 co-culture on transwells in presence of neutrophils (NEU), CD8 or neutrophils/CD8 (1:1 ratio) isolated from HD (n=2, relative to Figure 6G). DAPI blue, LD (dead cells) in yellow. Scale bar 100μm. **C.** Quantification of DAPI positive area in the fields relative to Figure 6H. **D.** Representative images of caspase staining (red) by multicolor confocal imaging on CACO-HT29 co-culture on transwells treated with PBS (CTR) or recombinant active human GZMK, with (GZMK+INHIBIT) or without (GZMK) the PRO-321 GZMK's inhibitor (24h). Scale bar 100μm.

Marker	Fluorophore	Reactivity	Manufacturer	Cat. #	Dilution
CCR7	BV421	Human	BD	562555	1:100
CD10	APCR700	Human	BD	659120	1:100
CD11b	BV786	Human	BD	740965	1:100
CD11C	APC R700	Human	BD	566610	1:100
CD127	APCR700	Human	BD	565185	1:100
CD14	BV421	Human	BD	565283	1:100
CD15	APC	Human	BD	551376	1:100
CD16	BV650	Human	BD	563692	1:100
CD19	BV650	Human	BD	563226	1:100
CD206	APC	Human	BD	550889	1:100
CD25	PE-CF594	Human	BD	562403	1:100
CD3	BB700	Human	BD	566575	1:100
CD33	BV421	Human	BD	562854	1:100
CD39	PECF594	Human	BD	563678	1:100
CD4	BV605	Human	BD	562658	1:100
CD45	APCH7	Human	BD	560178	1:100
CD45Ra	FITC	Human	BD	555488	1:100
CD66b	PE	Human	BD	561650	1:100
CD68	BV421	Human	BD	564943	1:100
CD69	APCCY7	Human	BD	560912	1:100
CD69	APC	Human	BD	555533	1:100
CD8	BV605	Human	BD	564116	1:100
CD8	BV786	Human	BD	563823	1:500
CD80	PE	Human	BD	557227	1:100
CTLA4	BV421	Human	BD	562743	1:100
CXCR2	BV421	Human	BD	744195	1:100
CXCR2	FITC	Human	BioLegend	320704	1:100
CXCR4	PECY7	Human	BD	560669	1:100
GRZK	PE	Human	Santa Cruz Biotechnology	sc-56125	1:100
HLA-DR	BV605	Human	BD	562845	1:100
LAG3	PE	Human	BD	565616	1:100
PD1	BV650	Human	BD	564104	1:100
TCR γ/δ	PE-CF594	Human	BD	562511	1:100
TIM3	BB515	Human	BD	565568	1:100
TCR γ/δ	PerCP-Cy5.5	Human	BioLegend	331224	1:10
NKG2A	FITC	Human	Miltenyi	130-113-568	1:100
CD39	APC-H7	Human	BioLegend	328226	1:50
TIGIT	APC	Human	BioLegend	372706	1:50
CD25	BV786	Human	BD	741035	1:600
CCR7	BV711	Human	BD	566602	1:20
OX40	BV650	Human	BD	563658	1:20
CD161	BV605	Human	Biolegend	339916	1:10
CD27	BV570	Human	BioLegend	302825	1:20
CD11b	BV510	Human	Biolegend	301334	1:10
PD1	BV480	Human	BD	566112	1:20
CD103	BV421	Human	BioLegend	350214	1:100
CD8	BUV805	Human	BD	564912	1:200
CD28	BUV737	Human	BD	564438	1:10
HLADR	BUV661	Human	BD	565073	1:100
CD4	BUV615	Human	BD	624297	1:400
CD45RA	BUV563	Human	BD	565702	1:100
CD3	BUV496	Human	BD	564809	1:20
CD69	BUV395	Human	BD	564364	1:100
CD45	PE-Cy7	Human	Biolegend	304016	1:2500
CD56	PE-CY5.5	Human	eBioscience	35-0567-42	1:20
CD127	PE-CY5	Human	eBioscience	15-1278-42	1:20
CX3CR1	PECF594	Human	Biolegend	341624	1:50
GZMB	APC-R700	Human	BD	560213	1:600
GZMK	PE	Human	Santa Cruz	sc-56125	1:200
Zombie		Human/Mouse	BioLegend	423102	1:800
Fixable Viability Stain	BV510	Human/Mouse	BD	564406	1:500

Supplementary Table 1

Marker	Fluorophore	Reactivity	Manufacturer	Cat. #	Dilution
CD45	PE CY7	Mouse	BioLegend	103114	1:200
Ly6G	APC CY7	Mouse	Tonbo	25-1276-U-025	1:200
CD16	FITC	Mouse	BD	553144	1:200
CXCR2	AF647	Mouse	BioLegend	149305	1:200
CD11B	PE	Mouse	eBioscience	12-0112-81	1:200
PD L1	BV421	Mouse	BD	564716	1:200
Ly6C	PeCF594	Mouse	BD	562728	1:200
CD8	BUV395	Mouse	BD	565968	1:200
CD45	BUV737	Mouse	BioLegend	748371	1:200
CD3	PerCP-Cy5.5	Mouse	BD	560527	1:200
CD103	FITC	Mouse	BD	557494	1:100
CD39	PECy7	Mouse	BioLegend	143805	1:200
NK1.1	EFLUOR780	Mouse	eBioscience	47-5941-80	1:200
CD25	BV650	Mouse	BD	564021	1:200
TIGIT	APC R700	Mouse	BD	565474	1:200
41BB	BV421	Mouse	BD	740898	1:200
CD28	PeCF594	Mouse	BD	562765	1:200
CD69	BV605	Mouse	BD	563290	1:200
CX3CR1	APC	Mouse	BioLegend	149008	1:200
PD1	BV785	Mouse	BioLegend	135225	1:200
GZMB	PB	Mouse	BioLegend	515407	1:100
GZMK		Human/Mouse	Invitrogen	PA5-50980	1:1000
CD66b		Human	BioLegend	305102	1:100
CD8	AF488	Human/Mouse	Invitrogen	53-0008-82	1:100
GZMK		Human/Mouse	Invitrogen	LS-C119554-50	1:100
ECAD		Human	Abcam	Ab1416	1:100
Ki67		Human/Mouse	eBioscience	SolA15	1:200
EPCAM		Human	Abcam	Ab32394	1:400
SDF-1		Human	R&D	MAB350	1:100
αSMA		Human	Abcam	Ab8211	1:200

Supplementary Table 1

List of antibodies for Flow Cytometry. Markers with the corresponding Fluorophore, Manufacturer, Catalog ID (Cat. #) and used dilution are shown.

T cell population	Gene Signature
CD8 Effector memory T cells (CD8_Tem)	CD3D, CD3E, CD8A, CD8B, GZMM, IFNG, GZMK, CCL5, MT-ND1, SRSF7, CXCR4, JUNB, CRTAM.
CD8 Effector memory T cells (CD8_Tem-GZMK^{high})	CD3D, CD3E, CD8A, CD8B, IFNG, GZMK, CCL5, MT-ND1, SRSF7, CXCR4, JUNB, CRTAM, TNFRSF9, CMC1, JUN, CCL3L1.
CD8 Effector memory T cells-HLADR CD8 (CD8_Tem-HLADR)	CD3D, CD3E, CD8A, CD8B, CCL5, JUNB, CRTAM, HLA-DRB5, HLA-DRA, HLA-DPB1, HLA-DRB1, GZMK, HLA-DQA1, CD74, ZFP36, CXCR4.
CD8 Effector memory T cells PD-1 (CD8_Tem-PD1)	CD3D, CD3E, CD8A, CD8B, GZMK, CCL5, MT-ND1, CXCR4, CRTAM, PDCD1.
CD8 Terminal effector T cells (CD8_Tte)	CD3D, CD3E, CD8A, CD8B, GNLY, FGFBP2, FCGR3A, GZMH, GZMB, TRGC2, PRF1, ZEB2, RORA, MT-ND3, FMNL1.
CD8 Exhausted T cells (CD8_Tex)	CD3D, CD3E, CD8A, CD8B, CXCL13, CD39, LAYN, CTLA4, RBPJ, DUSP4, GZMA, HAVCR2.
Naive-Like T cells (Naïve-like)	CD3D, CD3E, SELL, CCR7, IL7R, TPT1, RPS12, GPR183, RPS18, KLF2, LTB.
CD4 Regulatory T cells (Treg)	CD3D, CD3E, CD4, FOXP3, TNFRSF4, IL1R1, IL2RA, CCR6, TNFRSF18, BATF, CTLA4, CCL20, DNPH1, LTB, IL32.
CD4 Effector memory T cells (CD4_Tem)	CD3D, CD3E, CD4, JUNB, GZMK, GPR183, KLRB1, CTLA4, ICOS, STAT3, CD28, PDCD1.
Mucosal associated invariant T cells (MAIT)	CD3D, CD3E, CD4, CD40LG, IL7R, LTB, KLRB1.
Natural killer T cells (NK)	TYROBP, FCER1G, KLRF1, FGFBP2, FCGR3A, GNLY, KLRD1, TRDC, PRF1, NKG7, GZMB, IFNG.
Gamma delta T cells (Tgd)	CD3D, CD3E, TRDC, TRGC1.

Supplementary Table 2

Gene signatures used to annotate T cell subtypes.
