# Supplementary material for: GZMKhigh CD8+ T effector memory cells are associated with CD15high neutrophil abundance in non-metastatic colorectal tumors and predict poor clinical outcome
Source: Nat Commun. 2022 Nov 8;13:6752. doi: 10.1038/s41467-022-34467-3 (PMC9643357; doi:10.1038/s41467-022-34467-3)
Supplement: Supplementary file 2 — Reporting Summary [file 41467_2022_34467_MOESM2_ESM.pdf]

nature portfolio

Corresponding author(s): Luigi Nezi, Teresa Manzo

Last updated by author(s): 2022/10/12

Reporting Summary

Nature Portfolio wishes to improve the reproducibility of the work that we publish. This form provides structure for consistency and transparency in reporting. For further information on Nature Portfolio policies, see our Editorial Policies and the Editorial Policy Checklist.

Statistics

For all statistical analyses, confirm that the following items are present in the figure legend, table legend, main text, or Methods section.

n/a	Confirmed	
☐	☒	The exact sample size (*n*) for each experimental group/condition, given as a discrete number and unit of measurement
☐	☒	A statement on whether measurements were taken from distinct samples or whether the same sample was measured repeatedly
☐	☒	The statistical test(s) used AND whether they are one- or two-sided<br>*Only common tests should be described solely by name; describe more complex techniques in the Methods section.*
☐	☒	A description of all covariates tested
☐	☒	A description of any assumptions or corrections, such as tests of normality and adjustment for multiple comparisons
☐	☒	A full description of the statistical parameters including central tendency (e.g. means) or other basic estimates (e.g. regression coefficient) AND variation (e.g. standard deviation) or associated estimates of uncertainty (e.g. confidence intervals)
☐	☒	For null hypothesis testing, the test statistic (e.g. *F*, *t*, *r*) with confidence intervals, effect sizes, degrees of freedom and *P* value noted<br>*Give P values as exact values whenever suitable.*
☒	☐	For Bayesian analysis, information on the choice of priors and Markov chain Monte Carlo settings
☒	☐	For hierarchical and complex designs, identification of the appropriate level for tests and full reporting of outcomes
☐	☒	Estimates of effect sizes (e.g. Cohen's *d*, Pearson's *r*), indicating how they were calculated

Our web collection on statistics for biologists contains articles on many of the points above.

Software and code

Policy information about availability of computer code

Data collection	Flow cytometry data were collected using FACSymphony A5 or FACSCelesta with FACSDiva Software.<br>Sequencing data were collected using a NovaSeq 6000 System (Illumina).
Data analysis	FlowJo v10 and PhenoGraph version1.5 have been used to analyze flow cytometry data.<br>GraphPad Prism v_9.2.0 has been used to analyze the data.<br>scRNAseq data were analyzed using Seurat v. 3 R package<br>ImageJ version 2.0.0 has been used for confocal images.<br>Others: Microsoft Excel version 16.65, R software version 4.0.2.

For manuscripts utilizing custom algorithms or software that are central to the research but not yet described in published literature, software must be made available to editors and reviewers. We strongly encourage code deposition in a community repository (e.g. GitHub). See the Nature Portfolio guidelines for submitting code & software for further information.

Data

Policy information about availability of data

All manuscripts must include a data availability statement. This statement should provide the following information, where applicable:

- Accession codes, unique identifiers, or web links for publicly available datasets
- A description of any restrictions on data availability
- For clinical datasets or third party data, please ensure that the statement adheres to our policy

The transcriptomic data generated in this study have been deposited in the European Genome-Phenome Archive (EGA) under accession code EGAS00001006665. Code to reproduce Kaplan Meier plots of the manuscript can be found here (https://github.com/DanCag/survival-analysis). The transcriptomic data are available under restricted access in accordance with art. 13 General Regulation on Data Protection (EU Regulation 2016/679), access can be obtained by request to EGA and approval by the competent Data Access Committee. The TCGA-COAD and TCGA-LUAD gene expression data used in this study are publicly available in the GDC Data Portal (https://portal.gdc.cancer.gov/). Survival tables of TCGA-COAD and TCGA-LUAD cohorts were downloaded from cBioPortal (https://www.cbioportal.org/). The remaining data are available within the Article, Supplementary Information or Source Data file.

Human research participants

Policy information about studies involving human research participants and Sex and Gender in Research.

Reporting on sex and gender	Sex information were self-reported by patients from this study or retrieved from the public database of the TCGA cohorts. Gender details were not available. All the cohorts used in this study were sex balanced. All the cohorts used in this study were sex balanced, thus findings apply to both sex. Composition of our cohor of study is presented in Table 1 with data disaggrated based on sex. Sex inofmation has been reported in the source data when needed.
Population characteristics	Tumors, normal adjacent tissue and peripheral blood samples from 46 patients diagnosed with CRC were utilized in this study. Further characteristics of the cohort are provided in Table 1 of the manuscript.
Recruitment	All patients that were diagnosed with CRC non-metastatic and treatment-naïve at the time of surgical resection. All samples were collected at European Institute of Oncology (IEO).
Ethics oversight	The use of human samples was approved by the European Institute of Oncology (IEO) Institutional Review Board (protocol n. R1083/19-IEO 1149). All donors provided written informed consent in accordance with the Declaration of Helsinki. Samples were numerically coded to protect donor's rights to confidentiality and privacy.

Note that full information on the approval of the study protocol must also be provided in the manuscript.

Field-specific reporting

Please select the one below that is the best fit for your research. If you are not sure, read the appropriate sections before making your selection.

☒ Life sciences ☐ Behavioural & social sciences ☐ Ecological, evolutionary & environmental sciences

For a reference copy of the document with all sections, see nature.com/documents/nr-reporting-summary-flat.pdf

Life sciences study design

All studies must disclose on these points even when the disclosure is negative.

Sample size	Sample size was determined considering the mean of the target values between the different experimental groups, the standard error and the statistical analysis used. Moreover, sample size was determined based on previous experience in the lab which allow for statistical comparison. For cell data, we collected data form three biological replicates when possible.
Data exclusions	No data were excluded, except for three scRNA-seq samples due to cell numbers. However, the whole dataset is available.
Replication	All in vitro experiments were repeated at least two times and they were successfully reproduced. All in vivo mouse data were performed two times and the data were reproducible between experiments. The experiment with neutrophil depletion in vivo was performed once.
Randomization	For in vivo tumor experiments, mice were randomized prior to treatment. For all the other experiments, healthy donor and patients were randomly selected in order to avoid potential biases.
Blinding	For human samples, when possible, the order of acquiring the sample during imaging and flow cytometry was hidden for the investigator during the acquisition of all samples and the gating of results. The the samples were identified into groups at the stage of entering the data into the statistical package. For mouse experiments, investigators were not blinded during data collection and analysis because there were not involving subjective measurements.

Reporting for specific materials, systems and methods

We require information from authors about some types of materials, experimental systems and methods used in many studies. Here, indicate whether each material, system or method listed is relevant to your study. If you are not sure if a list item applies to your research, read the appropriate section before selecting a response.

Materials & experimental systems

n/a	Involved in the study
☐	☒ Antibodies
☐	☒ Eukaryotic cell lines
☒	☐ Palaeontology and archaeology
☐	☒ Animals and other organisms
☒	☐ Clinical data
☒	☐ Dual use research of concern

Methods

n/a	Involved in the study
☒	☐ ChIP-seq
☐	☒ Flow cytometry
☒	☐ MRI-based neuroimaging

Antibodies

Antibodies used

CCR7 BV421 Human BD 562555 1:100
CD10 APCR700 Human BD 659120 1:100
CD11b BV786 Human BD 740965 1:100
CD11C APC R700 Human BD 566610 1:100
CD127 APCR700 Human BD 565185 1:100
CD14 BV421 Human BD 565283 1:100
CD15 APC Human BD 551376 1:100
CD16 BV650 Human BD 563692 1:100
CD19 BV650 Human BD 563226 1:100
CD206 APC Human BD 550889 1:100
CD25 PE-CF594 Human BD 562403 1:100
CD3 BB700 Human BD 566575 1:100
CD33 BV421 Human BD 562854 1:100
CD39 PECF594 Human BD 563678 1:100
CD4 BV605 Human BD 562658 1:100
CD45 APCH7 Human BD 560178 1:100
CD45Ra FITC Human BD 555488 1:100
CD66b PE Human BD 561650 1:100
CD68 BV421 Human BD 564943 1:100
CD69 APCCY7 Human BD 560912 1:100
CD69 APC Human BD 555533 1:100
CD8 BV605 Human BD 564116 1:100
CD8 BV786 Human BD 563823 1:500
CD80 PE Human BD 557227 1:100
CTLA4 BV421 Human BD 562743 1:100
CXCR2 BV421 Human BD 744195 1:100
CXCR2 FITC Human BioLegend 320704 1:100
CXCR4 PECY7 Human BD 560669 1:100
GRZK PE Human Santa Cruz Biotechnology sc-56125 1:100
HLA-DR BV605 Human BD 562845 1:100
LAG3 PE Human BD 565616 1:100
PD1 BV650 Human BD 564104 1:100
TCR γ/δ PE-CF594 Human BD 562511 1:100
TIM3 BB515 Human BD 565568 1:100
TCR γ/δ PerCP-Cy5.5 Human BioLegend 331224 1:10
NKG2A FITC Human Miltenyi 130-113-568 1:100
CD39 APC-H7 Human BioLegend 328226 1:50
TIGIT APC Human BioLegend 372706 1:50
CD25 BV786 Human BD 741035 1:600
CCR7 BV711 Human BD 566602 1:20
OX40 BV650 Human BD 563658 1:20
CD161 BV605 Human Biolegend 339916 1:10
CD27 BV570 Human BioLegend 302825 1:20
CD11b BV510 Human Biolegend 301334 1:10
PD1 BV480 Human BD 566112 1:20
CD103 BV421 Human BioLegend 350214 1:100

CD8 BUV805 Human BD 564912 1:200
CD28 BUV737 Human BD 564438 1:10
HLADR BUV661 Human BD 565073 1:100
CD4 BUV615 Human BD 624297 1:400
CD45RA BUV563 Human BD 565702 1:100
CD3 BUV496 Human BD 564809 1:20
CD69 BUV395 Human BD 564364 1:100
CD45 PE-Cy7 Human Biolegend 304016 1:2500
CD56 PE-CY5.5 Human eBioscience 35-0567-42 1:20
CD127 PE-CY5 Human eBioscience 15-1278-42 1:20
CX3CR1 PECF594 Human Biolegend 341624 1:50
GZMB APC-R700 Human BD 560213 1:600
GZMK PE Human Santa Cruz sc-56125 1:200
Zombie Human/Mouse BioLegend 423102 1:800
Fixable Viability Stain BV510 Human/Mouse BD 564406 1:500
CD45 PE CY7 Mouse BioLegend 103114 1:200
Ly6G APC CY7 Mouse Tonbo 25-1276-U-025 1:200
CD16 FITC Mouse BD 553144 1:200
CXCR2 AF647 Mouse BioLegend 149305 1:200
CD11B PE Mouse eBioscience 12-0112-81 1:200
PD L1 BV421 Mouse BD 564716 1:200
Ly6C PeCF594 Mouse BD 562728 1:200
CD8 BUV395 Mouse BD 565968 1:200
CD45 BUV737 Mouse BioLegend 748371 1:200
CD3 PerCP-Cy5.5 Mouse BD 560527 1:200
CD103 FITC Mouse BD 557494 1:100
CD39 PECy7 Mouse BioLegend 143805 1:200
NK1.1 EFLUOR780 Mouse eBioscience 47-5941-80 1:200
CD25 BV650 Mouse BD 564021 1:200
TIGIT APC R700 Mouse BD 565474 1:200
41BB BV421 Mouse BD 740898 1:200
CD28 PeCF594 Mouse BD 562765 1:200
CD69 BV605 Mouse BD 563290 1:200
CX3CR1 APC Mouse BioLegend 149008 1:200
PD1 BV785 Mouse BioLegend 135225 1:200
GZMB PB Mouse BioLegend 515407 1:100
GZMK Human/Mouse Invitrogen PA5-50980 1:1000
CD66b Human BioLegend 305102 1:100
CD8 AF488 Human/Mouse Invitrogen 53-0008-82 1:100
GZMK Human/Mouse Invitrogen LS-C119554-50 1:100
ECAD Human Abcam Ab1416 1:100
Ki67 Human/Mouse eBioscience SolA15 1:200
EPCAM Human Abcam Ab32394 1:400
SDF-1 Human R&D MAB350 1:100
αSMA Human Abcam Ab8211 1:200

Validation

All antibodies used in our study are commercially available and validated. Antibodies were all titrated to determine the optimal concentration. Any further informations on the validation performed by the manufacturer can be retrieved at their website. All antibodies were first confirmed with their specific staining using known positive and negative cells with expected pattern.
Mouse antibody anti-GZMK was validated in house by: 1. titration to determine the optimal concentration by serial dilution (1:20; 1:50; 1:100; 1:200; 1:400; 1:800; 1:1000); 2. validation of its specificity by integrating critical control (I.e. negative and positive control, internal negative control - population of cells that do not express GZMK - FMO and unstained controls); 3. fixation and permeabilization protocol was also optimized.

Eukaryotic cell lines

Policy information about cell lines and Sex and Gender in Research

Cell line source(s)

Caco-2 were obtained from American Type Culture Collection (ATCC). HT-29 MTX cells were provided by Dr. Monteleone. Human Microvascular Endothelial Cells (HMEC-1) were obtained from Center for Disease Control and Prevention (CDC) of Atlanta. MC38 cell line was a gift from Dr. Chih-Hao Chang lab at The Jackson Laboratory.

Authentication

None of the cell lines used in this study were authenticated. However, the cell lines were obtained from the original source and behaved as expected in vitro and in vivo and this behavior did not change. Moreover, only low passage number cell line were utilized in this study.

Mycoplasma contamination

Cells were regularly tested negative for Mycoplasma ssp. by PCR analysis in-house. C

Commonly misidentified lines (See ICLAC register)	No commonly misidentified lines were used.

Animals and other research organisms

Policy information about studies involving animals; ARRIVE guidelines recommended for reporting animal research, and Sex and Gender in Research

Laboratory animals	Mice were housed and bred in a specific-pathogen-free animal facility in individually ventilated cages between 19-23C with 45-65% humidity and a 12 hour dark/light cycle. For mouse experiments, we used age-matched (10 weeks) C57BL/6J male mice in each experiment.
Wild animals	No wild animals were used in this study.
Reporting on sex	There was no sex biased in the animal studies used in this study.
Field-collected samples	No field-collected samples were employed in this study.
Ethics oversight	Animal handling and experimental protocols were reviewed and approved by the Institutional Animal Care and Use Committee of The Jackson Laboratory under the protocol number AUS#17027.

Note that full information on the approval of the study protocol must also be provided in the manuscript.

Flow Cytometry

Plots

Confirm that:

- [x] The axis labels state the marker and fluorochrome used (e.g. CD4-FITC).
- [x] The axis scales are clearly visible. Include numbers along axes only for bottom left plot of group (a 'group' is an analysis of identical markers).
- [x] All plots are contour plots with outliers or pseudocolor plots.
- [x] A numerical value for number of cells or percentage (with statistics) is provided.

Methodology

Sample preparation	High-dimensional flow cytometry was performed on T, NAT and PB (Brummelman et al., 2019). Conjugated antibodies used for flow cytometry are shown in Table 2. Briefly, single-cell suspensions were stained fresh or after thawing in pre-warmed RPMI with 10% FBS. Cells were washed in staining buffer (PBS with 2%FBS and 2mM EDTA) and incubated for 30min at 4°C with surface antibody cocktail in staining buffer, washed twice in staining buffer, fixed and permeabilized using Fixation/ Permeabilization Solution Kit (BD, Cat # 554714), and stained intracellularly for 1h at 4°C with an antibody cocktail in the permeabilization solution. Samples were acquired using a FACSymphony A5 or a FACSCelesta BVYG equipped with FACSDiva software version 8.0.1 (all from BD Biosciences). Compensation Beads (ThermoFisher, Cat# 01-2222-41) were used to prepare single-stained controls for electronic compensation. Dead cells were excluded using Fixable Viability Stain (BD). To evaluate ex vivo cytokines production, CD8+ T cell subsets were sorted to high purity using FACSAria III (BD Biosciences), then stimulated 3h with PMA (20ng/mL), ionomycin (1ug/mL) and GolgiPlug protein-transport inhibitor (brefeldin A, 1:1000).
Instrument	BD Bioscience CANTO I and BD Bioscience CANTO II and BD Bioscience Fortessa
Software	Flowcytometry data were collected with BD FACSDIVA software and analyzed and compensated with FLOWJO (V10) software.
Cell population abundance	Post-sort fractions were considered pure when the subset of interest was >90% of the sample. purity was evaluated by FACS acquisition immediately after sorting.
Gating strategy	Flow cytometric gating strategy for the isolation of neutrophils is indicated in Figure 1. Multicolor panels were tested and optimized first using FMO. Thus, after doublets and dead cells exclusion, neutrophils were selected as follow CD45+, CD56-, HLA-DR-, CD11b+, CD33dim, CD66b+ and then selected the CD15+ and CD15- populations applying NK cells (defined as CD11b+, CD66b-, CD56+) and M-MDSC (defined as CD11b+, HLA-DR-, CD66b-, CD33+) as negative biological controls for CD15 expression. CD8 analysis was performed on 3000 events per sample after manual gating isolation of singlet, LD negative, CD45+CD3+CD8+ T cells.

- [x] Tick this box to confirm that a figure exemplifying the gating strategy is provided in the Supplementary Information.
